# Detection of rare medical events in electronic health records using machine learning: Current practices and suggestions – A scoping review

**DOI:** 10.1371/journal.pone.0332963

**Published:** 2026-03-16

**Authors:** Biniyam Gebeyehu, Bennett Kleinberg, Katrijn Van Deun, Esther de Vries

**Affiliations:** 1 Tranzo, Tilburg School of Social and Behavioral Sciences, Tilburg University, Tilburg, The Netherlands; 2 Department of Methodology and Statistics, Tilburg School of Social and Behavioral Sciences, Tilburg University, Tilburg, The Netherlands; 3 Jeroen Bosch Academy Research, Jeroen Bosch Hospital, ‘s-Hertogenbosch, The Netherlands; 4 Laboratory of Medical Microbiology and Immunology, Elisabeth-Tweesteden Hospital, Tilburg, The Netherlands; Duke University, UNITED STATES OF AMERICA

## Abstract

**Background:**

Routine healthcare data are increasingly stored in electronic health records (EHRs), presenting an exciting opportunity to leverage machine learning (ML) for detecting and predicting medical events. While medical experts are optimistic about expanding its applications, several caveats exist which are often overlooked. Many medical outcomes are categorical (e.g., a diagnosis is present or absent) with categories being considerably unequal in size, which might significantly impact the performance of ML algorithms. Detecting small subgroups in EHR data, so-called anomaly detection, is an emerging approach, yet organized documentation on current practices remains scarce. This scoping review examines medical anomaly detection based on routine healthcare data stored in EHRs and formulated alternative approaches in case suboptimal practices were noticed.

**Methods:**

PubMed and Web of Science were searched up to September 5, 2024. Peer-reviewed articles and conference papers on ML-based medical anomaly detection in EHR data were included. Fifty-two study characteristics were extracted and analyzed both quantitatively and qualitatively.

**Results:**

A total of 117 studies met the inclusion criteria. The cross-study median proportion of the anomalous class was 0.079 (range 0.00045–0.23). Key details, e.g., data preprocessing actions, were often incomplete; 14.5% (n = 17) provided no information on this aspect. Only four studies reported the underlying cause of missingness before deciding how to handle it, and just three considered the clinical implications of false positives and false negatives when evaluating anomaly detection performance.

**Conclusion:**

We identified a need for greater attention in the current medical anomaly detection literature for reporting details on pre-processing, handling of missing data, and the use of performance metrics. With the increasing number of anomaly detection studies based on routine healthcare data stored in EHRs, more focus is needed on implementation and reporting practices to ensure relevance and reproducibility of future studies in this field.

## 1. Introduction

These days, routine healthcare data are recorded and stored digitally in electronic health records (EHRs). This has resulted in a vast and rapidly expanding repository of patient data, including, e.g., diagnoses, histories, laboratory results, interventions, and medications. This wealth of data has paved the way for a wide range of innovations, particularly through the application of machine learning (ML), e.g., [1,2]. Early and current applications of ML have been proven to be successful in supporting various clinical tasks, including disease diagnosis [3], early detection of adverse drug reactions (ADRs) [4], and identifying underdose or overdose prescriptions [5], which sparked optimism for broader implementation.

The application of machine learning (ML) in healthcare comes with certain caveats. When predicting categorical outcomes such as the presence or absence of a diagnosis or ADR, the effectiveness of machine learning algorithms depends on availability of comparable sample sizes across each category. However, in practice, the distribution of these outcomes is often imbalanced (asymmetrical) i.e., with the number of patients harboring a certain outcome (the minority class) being considerably smaller than the number of patients without (the majority class). This is most pronounced in the case of rare medical events [6]. The detection of such small subgroups is generally called ‘anomaly detection’. Considering how often uncommon medical events occur, successful implementation of anomaly detection based on routine healthcare data stored in EHRs could enhance a broad range of clinical tasks.

Anomaly detection already holds a significant role in application areas such as intrusion detection and financial fraud detection. This experience is reflected in the literature in those domains, where various anomaly detection approaches on data level (such as undersampling of the majority class and oversampling of the minority class) and on algorithmic level (such as cost-sensitive learning, i.e., taking the costs of prediction errors into account) are proposed [7–11]. This knowledge should be and increasingly is being used to develop anomaly detection in the medical domain. Examples are anomalies in chest radiography images [12], sleep apnea and respiratory anomaly detection [13], behavior monitoring in independently living elderly [14], and clinical decision support systems to monitor the health of critically ill patients in ICUs [15].

There are many so-called ‘performance metrics’ available for measuring the success of ML-based predictions such as specificity, sensitivity, and area under the receiver operating characteristic curve (ROC AUC), which are metrics that are already often used in the medical literature also when unrelated to ML-based anomaly detection, and many others (see below). Accuracy is often used in ML as a standard first metric. For anomaly detection this is an issue, as any algorithm focusing on the majority class will predict most cases correctly by always choosing that class (which is by far the most likely outcome), resulting in excellent accuracy, but far too many false negatives. In medical anomaly detection, these ‘false negatives’ are un- or misdiagnosed patients being under- or mistreated and potentially suffering from decreased quality of life and unnecessary complications (with accompanying healthcare and societal costs). On the other hand, if algorithms are specifically trained to catch every case in the minority class, this will lead to a huge increase in false positives, a phenomenon known as the false positive paradox [4], leading to unjustified extra, often costly investigations and unnecessarily worried patients. Finding the right balance is a challenge [5].

As medical anomaly detection based on EHR data continues to emerge as a key approach, with a further growing number of papers expected in the future, it is crucial to document current practices and assess their suitability. The current literature reveals only limited organized information on this topic. Besides, available medical anomaly detection studies vary in focus, being either predominantly medically or methodologically oriented, and are conducted by authors with diverse backgrounds, with the influence of these factors remaining unexplored in the current literature. Therefore, we conducted a comprehensive scoping review of the currently available medical literature on this topic, and propose alternatives where suboptimal practices were identified. We believe that this review will enable doctors and medical researchers to have a better picture of existing and preferred practices and will also help them to make better decisions when the application of medical anomaly detection is required in their future work.

We developed the following main research question: *which methods are used in ML-based medical anomaly detection studies, and what suboptimal practices (if present) do researchers need to be aware of?* To explore the answer to the main research question, we formulated four sub-questions regarding the included articles:

(1) What are the characteristics of the datasets being used in medical anomaly detection studies based on EHR data?
(2) What data preprocessing actions were taken and was there consistency in their reporting?
(3) Which algorithms (individually and by category) were applied, and which metrics were used to evaluate the results?
(4) What suboptimal practices were identified in the current medical anomaly detection studies based on EHR data, what should be done to avoid them, and are they influenced by the focus of the study (medical or methodological) and the authors’ backgrounds?

## 2. Methods

A brief definition of the technical terms used in this paper is given in Table 1.

**Table 1:** a brief explanation of the technical terms.

Term	Brief explanation
Anomalous class and normal class	The anomalous class encompasses outcomes that are rarely observed (less likely to be observed) or outcomes that do not conform to the expected pattern. The normal class represents the typical outcomes (outcomes we expect most often) or outcomes that conform to the expected pattern.
Anomaly detection	The process of identifying data points, events, or outcomes, that deviate from standard or expected behavior.
Biologically implausible values	Values that are not realistic or beyond the scientifically acceptable range for a given measurement.
Clustering-based (unsupervised) algorithms	A class of unsupervised algorithms that are based on the assumption that anomalies either belong to a small sparse cluster (containing relatively few data points) or do not belong to any cluster at all.
Categorical variable	A variable that represents data that can be divided into groups or categories, such as blood groups (A, B, AB, O).
Classification-based (unsupervised) algorithms	A class of unsupervised anomaly detection that learns the region occupied by normal data and defines a boundary around it, and labels any data point falling outside this boundary as anomalous.
Cross-validation	A technique that involves dividing the dataset into multiple parts (also called folds) and training the machine learning algorithm on some parts and evaluating the trained algorithm on the remaining part(s).
Data preprocessing	Data preprocessing is a series of operations (such as outlier detection, scaling/normalization, and missing imputation) aimed at converting the raw data into a format suitable for the training of a machine learning algorithm.
Data split	A data split or train-test split is the partitioning of a dataset into subsets for training and evaluating machine learning algorithms separately.
Distance	A function that quantifies how far apart two data points are.
Distributional assumption	Best guessing or assuming how data behaves (such as the function that generated the data, mean, and standard deviation), and when such assumptions are made the result might not be valid unless the assumptions hold.
Ensemble method	A process by which multiple algorithms, such as several supervised machine learning algorithms, are systematically applied and combined mainly for improving the generalizability and performance of the prediction model.
Feature (variable) selection	This refers to the process of obtaining a subset of features (and getting rid of irrelevant ones) from an original feature (variable) list according to certain criteria. Examples of feature selection (adopted by studies included in the review): area under curve (AUC)-based, chi-squared-based, data completeness, dragonfly algorithm, evolutionary search (ES), embedded method, feature selection via supervised model construction (FSSMC), forward selection, group sparsity, impurity-based, knowledge and data combined feature selection (KDFS), lasso regression, literature-based, map reduce-based machine learning algorithms (MR-PB-PFS), mean comparison, minimum redundancy maximum relevance (mRMR), particle swarm optimization (PSO), relief, recursive feature elimination, sensitivity analysis, stepwise section, and wrapper method.
Hyperparameters	Set (range) of values that control how a machine learning algorithm learns and performs.
Hyperparameter tuning	Is the process of finding the best hyperparameter values for a machine learning algorithm so it can perform as well as possible.
Machine learning	Machine learning is a tool designed to learn patterns from a dataset. Machine learning uses algorithms, i.e., a process or set of rules to be followed in calculations or other problem-solving operations. An algorithm needs input variable(s) (also called features, independent variables, or predictors). An algorithm can predict a pre-defined output variable (outcome variable, dependent variable, or label). Machine learning can be broadly classified into supervised learning (the machine learning algorithm is based on a dataset that contains a label), unsupervised (the machine learning is based on only input variables, the dataset does not contain a label, or it is not used), or semi-supervised (machine learning based on a partially labeled dataset).
Missing data imputation	Missing data imputation is the process of substituting the missing values in the dataset with a reasonable, estimated alternative to the missed value. Examples of imputation techniques used: K-means, Hierarchical Gaussian processes (HGPs), random forest (RF) regressor, Mean, k-nearest neighbor (k-NN), mode, a random forest–based imputation algorithm (missForest), multivariate imputation by chained equations (MICE), median, spline interpolation.
Missing value	Missing values or missing data are subsets of the data for which values are not available or cannot be measured.
Missingness mechanism	A process by which part of the data becomes missing. It characterizes how the missing data are related to the features (variables) in the dataset. There are three missingness mechanisms: (1) Missing completely at random (MCAR): the reason why some data is missing is unrelated to the information we already have or any other factor in the study, or the chance of data being missing is the same for every individual in the cohort. (2) Missing at random (MAR): the reason why some data is missing is related to other information we already have, but not to the missing data itself. (3) Missing not at random (MNAR): the reason why some data is missing is related to the missing data itself.
NN-based (unsupervised) algorithms	A class of unsupervised algorithms that are based on the assumption that anomalous data points lie relatively far from their closest neighbors while normal data lie close to each other.
Non-parametric methods	A set of statistical methods that do not make assumptions about the underlying probability distribution of the data.
One-class learning	A type of machine learning where the machine learning algorithm is expected to learn only the pattern in the normal class and use the learned pattern to identify data points that do not belong to the normal class (anomalous class).
Outlier detection	Outlier detection is a process of identifying and handling data point(s) that deviate significantly from the majority of the data points. Examples of outlier detection techniques used by the studies included: artificial bee colony (ABC), density-based spatial clustering of applications with noise (DBSCAN), isolation forest (iForest), local outlier factor (LOF), K-means, one class support vector machine (OC-SVM), visualization techniques such as boxplot, and z-distribution.
Performance metrics	Performance metrics are measures that show how close the predicted and actual values are or how well a given machine learning algorithm performs in predicting unknown outcomes.
Resampling	Resampling is a process of generating new data or removing part of the data to balance the sample sizes (number of observations) across categories. Several resampling techniques are available in the literature such as oversampling (duplicating selected data points from the minority class) and undersampling (removing selected data points from the majority class). Studies included in this review applied four different resampling techniques: undersampling, oversampling, synthetic minority oversampling technique (SMOTE), and synthetic minority oversampling technique with Tomek Links (SMOTEtomek).
Scaling/normalization	A technique in which values of a certain variable are shifted and rescaled so that they end up ranging within certain ranges such as 0 and 1.
Statistical-based (unsupervised) algorithms	A class of unsupervised anomaly detection that first defines the statistical properties (based on measures such as mean and standard deviation) of the normal data points (the expected behaviors) and flag data points as anomalous that deviate significantly from the expected behaviors.
Statistical power	Measures the likelihood that a study can distinguish an actual effect or a true difference from a chance occurrence.
Stratified split	Is a technique used to ensure that the distribution of classes in the dataset is preserved in each subset (e.g., training, validation, and test sets).
Subspace based (unsupervised) algorithms	A class of unsupervised anomaly detection that focuses on finding anomalous data points in a subgroup of the full data. These methods are specifically applicable in the presence of many features (variables), as anomalous data points might appear normal when considering the entire dataset, but their anomalousness is revealed when considering a combination of features (subspaces).
Unbalanced data (class imbalance)	In supervised machine learning algorithms, the data is unbalanced when the outcome variable is categorical (the outcome has distinct categories) and the number of samples under each category is far from equal (some of the categories are significantly more frequent than others).

### 2.1. Approach

As the main aim of this study is to map existing medical anomaly detection studies based on EHR data in the literature and identify suboptimal practices, we opted for the scoping review methodology [16]. The review was done according to a set of recommendations provided by Arksey and O’Malley which has been further refined by Levac et al [17]. We applied the first five stages of this six-stage methodological framework: 1) identifying the research question; 2) identifying relevant studies; 3) study selection; 4) charting the data; 5) collating, summarizing, and reporting the results. For reporting, we have used the Preferred Reporting Items for Systematic Reviews and Meta-Analyses: Extension for Scoping Reviews (PRISMA-ScR) guidelines [18]. The completed PRISMA-ScR checklist can be found in S1 Table.

### 2.2. Information sources

Based on the research questions, studies eligible for inclusion should meet the following criteria: the focus of interest of the study is a medical event; the manuscript has to explicitly indicate the anomaly detection approach used, present the anomaly detection results, be published in a peer-reviewed journal or be a peer-reviewed conference paper, be published in English, and be available in full text. We excluded studies that were based on visual and audio data, and data generated from devices such as wearable sensors.

### 2.3. Search strategy

We conducted a systematic search of PubMed and Web of Science based on these criteria on September 5, 2024, without time restriction. The search strategy was developed around the two major features of the scoping review - ‘anomaly detection’ and ‘medical’. Keywords and keyword phrases were developed for the two major features and then combined using the ‘AND’ operator. First, we developed a search query for PubMed and then adapted the query to Web of Science. Keywords used to include or exclude studies based on data source type (e.g., EHR-based, sensor-generated, etc.) were not included in the search strategy to allow the identification of studies that provided limited information about their data source during the search stage. The full search strategy is available in S2 Table. In addition to the studies identified through database search, additional studies were identified through snowball and citation search.

### 2.4. Selection of sources of evidence

We deduplicated the initial search results using Rayyan [19]. Then, a title screening was conducted based on the inclusion criteria. In the title screening, the first author (BG) labeled each study as “Include”, “Exclude”, or “Maybe”; the same was performed in a blinded manner by one senior author each for each third of the retrieved articles (BK, KVD, and EdV). Thereafter, we conducted abstract screening in a similar fashion for all articles which at least one author had labeled as “Include” or “Maybe” in the title screening. Finally, full-text screening was conducted by the first author (BG) for studies labeled “Include” or “Maybe” by at least one of the abstract reviewers. All undecided results in the full-text screening were discussed within the research team until consensus was reached.

### 2.5. Charting the data

Data abstraction was performed on the final set of included studies. First, a data collection/abstraction protocol was developed to systematically collect all relevant information, as listed in Table 2. Then, a pilot data abstraction was performed on five studies by the first author (BG). All authors together then discussed the pilot data abstraction to evaluate whether the information collected was correct and in accordance with the research questions of the scoping review until discrepancies were solved. Thereafter, the data extraction was finalized by the first author (BG).

**Table 2:** Information extracted from the included studies.

Topic	Extracted information
General information	authors, journal, volume, DOI, title, year
Dataset information & size	Data accessibility, dataset name, disease/health issue, study population, target population, number of patients (cases) (both before and after over/under sampling), number of features (excluding the number of labels for supervised learning) (both before and after feature selection), hospital, country, city
Anomaly-related	Name of the minority (anomalous) class, number of patients (cases) in the minority class (both before and after over/under sampling), name of the majority (normal) class, proportion of the minority (anomalous) class (both before and after under/over sampling)
Pre-processing and algorithms	Data split type, cross-validation type, detection of biologically implausible values, scaling/normalization, missing action, missing imputed (method), over/under sampling technique, feature selection method, name of ML algorithm, objective(s) of ML algorithm
Performance metrics	ROC AUC, PR AUC, F1-score, precision, accuracy, FNR, FPR, FP, FN, sensitivity, specificity, Youden, kappa, other metrics
Limitations	Data-related, method-related, application-related
Remarks	Main contribution(s) of the study

*DOI, Digital Object Identifier; FN, Number of false negatives; FNR, False Negative Rate; FP, Number of false positives; FPR, False Positive Rate; ML, Machine Learning; PR AUC, Area Under the Precision Recall Curve; ROC AUC, Area Under the Receiver Operating Characteristic Curve.*

### 2.6. Collecting, summarizing, and reporting results

The extracted dataset was summarized using both quantitative (e.g., percentages and frequency tables) and qualitative data analysis [20]. Categorical characteristics such as the name of the ML algorithm (k-nearest neighbors (kNN), support vector machine (SVM), logistic regression (LR), etc.) are presented using descriptive tools such as tables and graphs.

To describe preprocessing consistently across studies, we operationalized it as the presence of the following predefined steps before applying a ML algorithm: (1) data split in training and test sets, (2) detection of biologically implausible values, (3) scaling/normalization, (4) missing value handling, and (5) variable selection. The ML algorithms were first categorized into supervised (a labeled outcome variable is predicted), unsupervised (a pattern in the data is sought), and semi-supervised (a combination of both). To add more context to the approach unsupervised algorithms used to identify anomalous data points, we adopted a categorization defined in [21] and adapted by Goldstein & Uchida [22], with slight alteration. The six categories of unsupervised algorithms created were: nearest neighbor (NN)-based, clustering-based, subspace-based, statistical-based, classification-based, and other (see also below). The first four categories were adopted as they are and the last two categories were created by splitting the category ‘classification based/other’ in [22]. See S1 Text for the detailed description of the six categories of unsupervised algorithms.

## 3. Results

We present the results section in five parts. First, an overview of the literature search is presented (Section 3.1). Next, the characteristics of the datasets used are presented (Section 3.2), as well as the data processing actions described in the included articles (Section 3.3), and the resampling techniques, ML algorithm(s) used, and performance metrics reported (Section 3.4). The classification of studies by their focus and authors’ affiliations is presented in Section 3.5. Finally, suboptimal practices we noticed among the included studies are described (Section 3.6).

### 3.1. Overview of the search results

**Fig 1:**
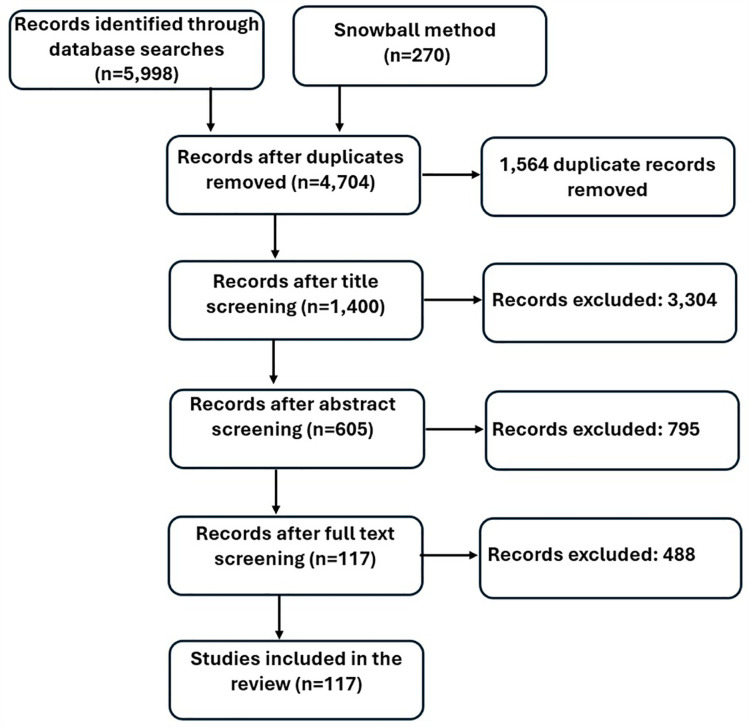
Flow diagram showing the study selection process.

**Fig 2:**
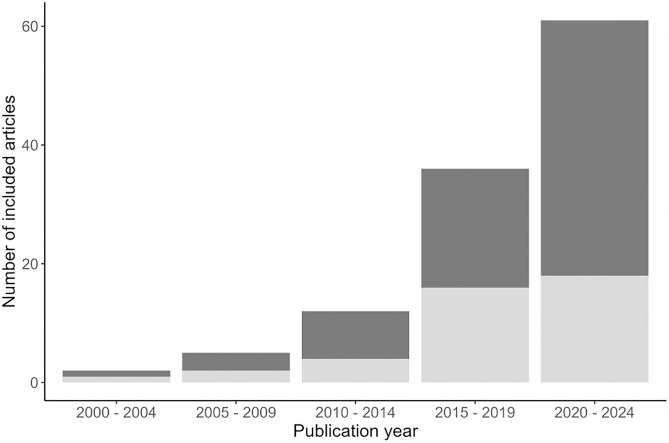
The number of medical anomaly detection articles based on EHR data by data accessibility. Dark grey = open, light grey = protected. Open = studies based on publicly accessible or accessible with request data (stated in the papers), protected = studies based on publicly inaccessible data (at the time of data extraction).

The literature search yielded 6,268 possibly relevant studies, including 5,998 from database searching and 270 from snowball and citation searching. After the removal of duplicates, 4,704 articles were assessed for eligibility based on consecutive title, abstract, and full-text screening, resulting in inclusion of 117 medical anomaly detection studies based on EHR data (details in Fig 1); the included studies showed an exponential increase in the course of time (Fig 2). The 117 studies included in this scoping review are listed in S3 Table.

### 3.2. Characteristics of the datasets used in the included studies

Study populations in the articles included in this review mainly originated in three countries: the United States (n = 44), Brazil (n = 13), and China (n = 12) (details in S4 Table), probably because of the availability of certain open datasets that are frequently used by researchers such as a dataset provided by Hospital Israelita Albert Einstein [23].

All included studies represented anomalous and normal data as a binary outcome, with non-binary outcomes (e.g., multiclass outcomes) converted through binary recoding. The median proportion of the anomalous (minority) class in the included studies was 0.079 (range 0.00045–0.23). In the majority of the datasets used by the included studies (n = 90; 77%), the class imbalance resulted from the natural frequency of events, such as the proportion of patients who experience adverse outcomes vs. those who do not [24]. In the remaining studies, the causes of the class imbalance were purposely undersampling one of the classes to create an artificial anomalous class for research purposes (n = 26, examples in [25]), or the definition of the classes (n = 7, e.g., length of hospital stay above vs. below a certain number of days [26]).

### 3.3. Data preprocessing

**Fig 3:**
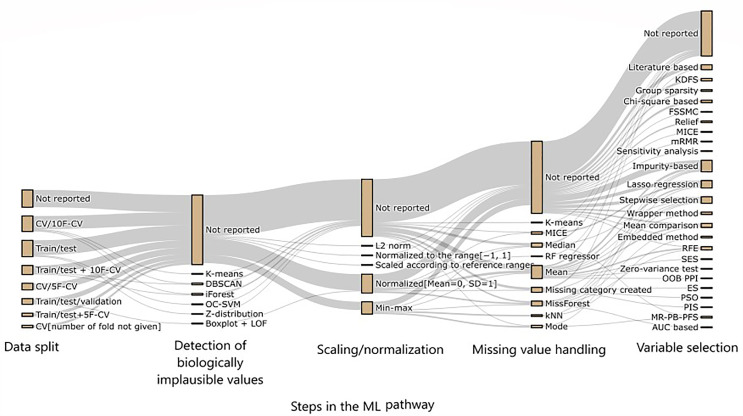
Data preprocessing actions described by the included studies. The figure presents the consecutive preprocessing actions from left to right: data split in training and test set (to be able to evaluate the performance of the algorithm on previously unseen data), detection of biologically implausible values (to be able to remove these from the dataset), scaling/normalization (to prevent bias due to the algorithm giving to much weight to variables with intrinsically larger numeric values), missing value handling (see methods and results), and variable selection (to use only those variables for predictions that have significant influence). ABC, Artificial bee colony; AUC, Area under the curve; CV, Cross validation, DBSCAN, Density-Based Spatial Clustering of Applications with Noise; ES, Evolutionary search; FSSMC, Feature selection via supervised model construction; HGPs, Hierarchical Gaussian processes, KDFS, Knowledge and data combined feature selection; k-NN, K-nearest neighbors, LOF, Local outlier factor; MICE, Multiple imputation by chained equations; *missForest, a random forest–based imputation algorithm;* ML, Machine learning; mRMR Minimum redundancy maximum relevance; MR-PB-PFS, Map reduce-based machine learning algorithms; OC-SVM, One class support vector machine; OOB PPI, Out-of-bag permuted predictor importance; PSO, Particle swarm optimization; REF, recursive feature elimination; RF, Random forest.

The data preprocessing actions described in the included articles were very heterogeneous. Five articles reported five preprocessing actions, 17 reported four or more, 48 reported three or more, 74 reported two or more, and 100 reported at least one preprocessing action. The remaining 17 studies did not report on preprocessing. The studies reported the following data preprocessing actions: data split (n = 82; 70%), detection of biologically implausible values (n = 12; 10%), scaling/normalization (n = 44; 38%), missing value handling (n = 56; 48%), and variable selection (n = 55; 47%) in various combinations (details in Fig 3). A complete list of preprocessing methods for each study can be found in S5 Table.

### 3.4. Machine learning applications

#### 3.4.1. Resampling.

Four different resampling techniques (creating new samples based on the observed samples) were applied in 25 of the included studies, with eight of them applying two or more techniques. SMOTE was used in twenty studies, followed by undersampling (n = 7), oversampling (n = 4), and SMOTETomek (n = 3). Seven of these studies compared the change in the performance of anomaly detectors before and after the data was balanced. Overall, applying resampling resulted in better detection of true positives and worse detection of true negatives.

#### 3.4.2. Reported performance metrics.

Seventeen different performance metrics were reported in the included studies to evaluate the performance of the anomaly detection algorithms: ROC AUC, accuracy, brier score (BS), F1 score, false negative rate (FNR), false positive rate (FPR), G-mean, Kappa, Matthews correlation coefficient (MCC), negative predictive value (NPV), area under the precision recall curve (PR AUC), precision, recall (=sensitivity), specificity, Youden’s index (YI), harmonic mean for subspace selection (HMSS), and balanced error rate (BER).

**Fig 4:**
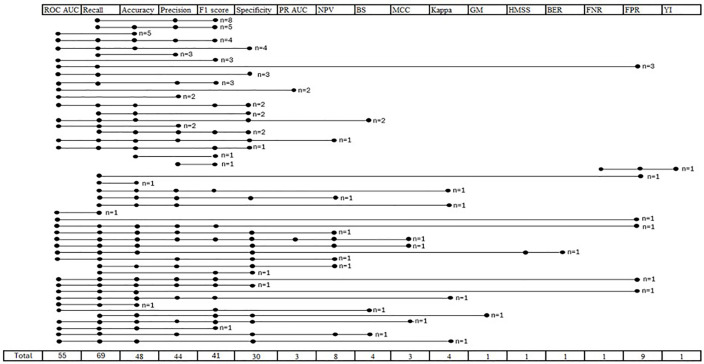
Performance metric co-occurrence among medical anomaly detection studies. The figure presents the co-occurrence of metrics when more than one performance metric was reported (each specific combination in a separate row, the respective metrics are shown in the column heads, n shows the number of studies with that specific combination). BS, Brier Score; FNR, False Negative Rate; FPR, False Positive Rate; MCC, Matthews Correlation Coefficient; NPV, Negative Predictive Value; PR AUC, Area Under the Precision Recall Curve*; ROC AUC, area under the receiver operating characteristic curve; YI, Youden’s index*.

Thirty-one studies reported one performance metric, being ROC AUC (n = 25), recall (n = 3), F1 score (n = 1), and PR AUC (n = 2). Eighty-six studies reported more than one metric (details in Fig 4).

#### 3.4.3. Machine learning algorithms.

**Fig 5:**
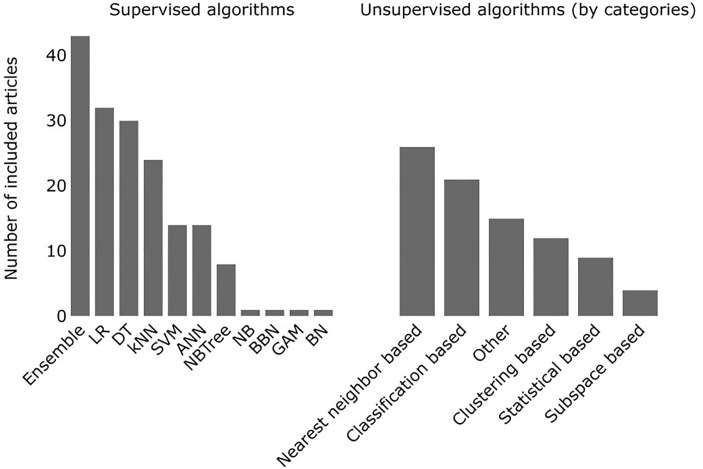
Frequency of used supervised algorithms (left) and unsupervised algorithm categories (right) in the included articles. ANN, artificial neural network; BBHA, binary black hole algorithm; DA, discriminant analysis; DT, decision tree; LR, logistic regression; NB, naïve Bayes; SVM, support vector machine.

Anomaly detection in the included studies involved 188 algorithms in total, categorized as supervised (n = 63), unsupervised (n = 52), or semi-supervised (n = 2) (Fig 5; details in S6 Table).

Ensemble methods, which combine two or more algorithms to enhance predictive strength, were the most frequently used (n = 40) in supervised machine learning. They also outperformed non-ensemble algorithms in most studies where both were applied. Among the five categories of unsupervised learning, NN-based algorithms were the predominant approaches (n = 25). This can be explained by their multiple strengths, such as not having assumptions regarding the distribution of the dataset, and intuitiveness: the anomalous data points are located far from their neighbors as determined by a distance metric. Among the studies that applied NN or clustering based algorithms, 23 reported a distance metric. The reported choices were Euclidean distance (n = 19), Mahalanobis distance (n = 3), and cosine similarity (n = 1). None of the studies compared multiple distance metrics to identify a better-performing option.

We assessed the relative performance of supervised algorithms used for the same task in the same study. Ensemble (combined) methods were compared to non-ensemble (single algorithms) methods 170 times. In 116 of these (68%), ensemble methods outperformed single algorithms based on ROC AUC scores. The least performing algorithm was decision tree (DT) e.g., out of the 18 studies that used both DT and logistic regression (LR), in 17 of them (94%) LR outperformed DT. Detailed information is presented in S7 Table.

### 3.5. Classification of studies by their focus and authors’ affiliations

Of the included studies, 62 (53%) focused on medical applications, while 55 (47%) focused on ML methodology. According to the authors’ backgrounds, studies were grouped as medical (n = 4; 3%), multidisciplinary/combination (n = 50; 43%), and methodological (n = 62; 53%). Medically focused studies provided more detailed information on the application of anomaly detection algorithms and evaluated their performance using a wider range of metrics; the median number of metrics reported by medically focused studies was 4, compared to 2 for methodologically focused studies (details in S8-S11 Tables).

### 3.6. Issues identified in the included studies

We identified three areas of potential suboptimal practices in the included studies, being the level of detail in reporting preprocessing steps, handling missingness, and choice of performance metrics.

We observed significant underreporting of preprocessing steps in the included studies. This was partly due to the fact that open source datasets were used, where preprocessing steps had already been reported in the original publications (n = 75; 64%). Among the 75 studies utilizing open datasets, 24 used datasets with documented preprocessing steps provided by the dataset provider. However, 11 of these studies did not acknowledge this preprocessing in their report. Specifically, seven studies omitted reporting on the handling of missing data, another seven failed to mention normalization or scaling processes, and two did not disclose assessments for biologically implausible values.

However, it also concerned articles describing studies on newly obtained datasets (n = 42; 36%). As an example, out of 117 studies included, only 44 reported whether the data was normalized/scaled, of which 14 concerned datasets that had not been described in a publication before. See also Section 3.3 for further details.

Out of the 117 included studies, 56 (48%) described whether missing data were present and how they had handled this. For the 61 remaining studies, it was not clear whether missing data were simply absent or what methods were applied to address them if they were present. Regarding the 56 studies that reported on their handling of missing data, 52 studies did not report on the mechanism of missingness; 19 (34%) performed a complete case analysis (they excluded cases with missing values) but without reporting an assessment of the potential consequences thereof.

There are dozens of possible metrics to evaluate the performance of machine learning algorithms, yet not all are suitable for anomaly detection. Regarding the use and reporting of metrics, we noticed three issues: (1) studies reported metrics that could be subject to misinterpretation in the presence of class imbalance (e.g., accuracy); (2) only four studies employed metrics that are considerate of the potential consequence of false positive and false negative predictions, the remaining studies relied on metrics such as ROC AUC (25 studies solely relied on it); (3) across studies, the same metrics are referred to by different names, e.g., F measure, F score, and F1 score were used interchangeably. Given that authors favor different names, reporting how they were calculated is essential for reader clarity.

## Discussion

This paper reports a scoping review on medical anomaly detection based on EHR data. Our aim was to conduct a comprehensive review of the currently available medical literature and to propose alternatives if suboptimal practices were identified. We could include 117 articles and conference papers. In supervised machine learning, ensemble methods were most frequently used, and they outperformed non-ensemble algorithms in most included studies where performance was compared within the same study, for the same task, and on the same dataset. However, in this study we did not aim to identify a single algorithm as the “best” or to draw definitive conclusions about the success of the applied anomaly detection algorithms, as the effectiveness of anomaly machine learning algorithms is context dependent and alternative algorithms may be more suitable for particular settings. Similar to other application domains [27], nearest neighbor-based algorithms were the predominant ones. That reliance on nearest neighbor-based approaches can be explained by their specific strengths, including not having assumptions regarding the distribution of the dataset, and intuitiveness (i.e., anomalous data points are those that are far from their neighbors as determined by a distance metric).

Prior medical research has highlighted concerns across various medical study categories, such as study bias [28], standard reporting [29], and ethical and integrity measures [30,31]. We identified several suboptimal practices in medical anomaly detection research on EHR data, which we discuss below. There was a marked increase in published medical anomaly detection studies based on EHR data in the past two decades. That increase underlines the importance of paying attention to the suboptimal practices we found to increase reproducibility, reliability, and the relevance of results to healthcare professionals who are generally not familiar with machine learning and its caveats when used for anomaly detection.

As described above, we identified three important caveats that were insufficiently addressed: (1) no or incomplete reporting of preprocessing steps, (2) failing to adequately address missing value issues, and, last but not least, (3) reporting of performance metrics that are inadequate for anomaly detection (or omitting discussion of their unreliability). Below, we discuss these three issues. We argue that future studies need to consider these issues while conducting medical anomaly detection studies.

When it comes to published articles, the written report is the sole medium of communication between researchers and their audience. As readers depend entirely on the written content, it is essential that all necessary methodological details are included. When essential details are missing, readers cannot adequately evaluate or replicate the work, reducing its scientific value. This issue is particularly concerning in rapidly growing fields (like medical anomaly detection based on EHR data), where incomplete reporting may set a poor precedent for future research. From the perspective of research integrity, failing to adequately report the methods of scientific research in any field is a form of questionable research practice as it delays discovery and understanding [32].

### Inadequate reporting of preprocessing steps

The issue of under-reporting key information is also noted in other types of medical studies, such as incompleteness of reporting in systematic reviews [33], in human genome epidemiology association studies [34], and in the functional neuroimaging literature [35]. Incomplete reporting of studies can have considerable consequences in the progress of any scientific field. Several solutions have been proposed, ranging from raising awareness about the value of complete reporting to developing standardized reporting guidelines [33,36]. The studies included in this review revealed significant underreporting of critical information in the analysis of medical anomaly detection based on EHR data. For instance, 17 studies (15%) provided no information on data preprocessing. This is partly due to studies based on existing open datasets that fail to report the preprocessing performed by the dataset provider. Notably, 11 of these studies either partially or completely omitted details of the preprocessing carried out by the original source.

### Handling missing data

The best strategy for handling missing data is to adopt a well-structured prospective study design and data collection process that minimizes the likelihood of missingness [37,38]. However, even among well-designed and carefully executed studies, missing data cannot always be avoided, also, missing data can *intrinsically* not be avoided when using routine healthcare data from the EHR (e.g., patients follow their own journeys through the hospital system). Several strategies have been developed to handle missing data such as only including participants/events without missing data (also known as complete case analysis [37,39]), imputation (estimating the missing values), and the missing indicator method (creating a new variable showing for each participant/event whether the variable under study has a value or not). The selection of an adequate approach to handle missing data mainly depends on the mechanism of missingness being MCAR, MAR, and MNAR (see above and Table 1). Identifying the mechanism of missingness is indispensable for deciding on the approach to handle missing data [40,41]. Complete case analysis is a common approach, and it is also the default approach in most statistical software packages [42]. However, this approach may result in biased findings if the missing data are not missing completely at random [43], on the other hand, it can be an effective strategy for supervised learning [44]. Anyway, discarding cases with missing data results in loss of statistical power [45]. In medical studies, it is generally unlikely that data are missing completely at random, especially when using EHR data [39] (e.g., healthier patients are more likely to have missing data such as blood sample results). In the included studies, 61 did not report on missingness, and 19 out of 56 that did address missing data used complete case analysis, but without reporting an assessment of the potential consequences thereof. Only 4 out of 56 reported whether they considered the mechanism of missingness. Studies that did address the missing data through imputation employed various methods such as the mean or median of the variable values in the dataset, or more complex algorithms (such as missForest, MICE) for estimating missing data. Several reviews are available that evaluate the current missing data handling techniques including for MNAR situations and provide guidance on selecting appropriate imputation.

### Choosing performance metrics

Performance metrics used in anomaly detection should be able to handle imbalanced datasets appropriately. While there are many metrics available to evaluate anomaly detection algorithms, there is no uniform agreement on which specific metric or combination of metrics should be used in which cases. Metrics like accuracy and error rate are not suitable to evaluate anomaly detection tasks, as they cannot handle the severe class imbalance. Such metrics should preferably not be presented, but when they are, authors should be cautious and explicitly address their limitations to avoid misleading the readers. There are studies that suggest a specific metric is preferred above one or more alternatives (e.g., ROC AUC [46], adjusted geometric-mean [47], PR AUC [48]). However, every metric has its drawbacks and cannot be used as ‘gold standard’ in every situation. E.g., F1 score can generate inflated overoptimistic results [49], and the most commonly used metric, ROC AUC (exclusively reported in 25 of the included studies), still does not show the clinical utility [50], because the clinical implications of false positives and false negatives are rarely equal, neither for the individual patients concerned, nor for the healthcare professionals and their organizations. Depending on the study objective, the limitations of relying solely on ROC can be addressed through several means such as by supplementing it with additional performance metrics [51], or by using approaches that quantify net clinical benefit [52]. Since no algorithm results in perfect prediction performance, the likely optimal cutoff point on a confusion matrix (a 2x2 table that summarizes the counts of correct and incorrect predictions) is determined by the context in which it is applied (for an example see S12 Table).

### Alternative approaches for suboptimal practices

For greater transparency, we recommend future studies include the list of features we propose in Table 3. Authors can incorporate these features, in addition to adopting a reporting guideline suitable for their study type and topic. Moving forward, we believe a standard reporting guideline for medical anomaly detection studies would be highly beneficial. Such a guideline would help authors adequately address class disparity while carrying out and reporting medical anomaly detection studies.

**Table 3:** A checklist of characteristics that are useful to report in publications on medical anomaly detection studies.

Study characteristics	Description
Name of the dataset	The name of the dataset used. This is specifically relevant when the dataset is publicly accessible as this helps to avoid confusion in cross-study comparisons of results and simplifies the search for detail on the dataset. For publicly available datasets, provide the source or access link.
Definition of the study population	Provide a description of the specific population group the study is based on, along with the inclusion and exclusion criteria. If the study involves sampling, describe the population from which the sample was drawn.
Outcome of interest	Provide a description of the variable(s) that the study aims to explain or predict (if applicable). If the outcome of interest is categorical, describe the categories. If the outcome is numeric but categorized, clearly describe the formed categories and the ranges for each category.
Anomalous group	Provide a description of the specific population group that represents the minority class (anomalous class).
Normal group	Provide a description of the specific population group that represents the majority class (non-anomalous class).
n-original	Present the total number of patients (cases) in the study. If some patients are excluded for specific reasons, provide the reasons for exclusion and the final number of patients (cases). If resampling is applied, this figure should reflect the number before resampling.
n-post-under/oversampling	Present the number of patients (cases) in the study after the application of resampling (if applicable).
n-anomaly-original	Present the number of patients (cases) in the minority class before resampling. If some patients (cases) are excluded for specific reasons, this figure should reflect the number after exclusion.
n-anomaly-post-under/oversampling	Present the number of patients (cases) in the minority class after resampling (if applicable).
P-anomaly-original	Present the proportion of the patients (cases) that belong to the anomalous class before the application of under/oversampling. This is calculated as n-anomaly-original divided by n-original.
P-anomaly-post-under/oversampling	Present the proportion of the patients (cases) that belong to the anomalous class after the application of under/oversampling (if applicable). This is calculated as n-anomaly-post-under/oversampling divided by n-anomaly-post-under/oversampling.
n-features-original	Provide the number of features (variables) before applying feature (variable) selection or dimensionality reduction. If some features are excluded during data preprocessing (other than feature selection), specify the number of features after preprocessing.
n-features-after-feature-selection	Provide the number of features (variables) after applying feature (variable) selection or dimensionality reduction (if applicable).
Feature selection	The name of the feature (variable) selection or the dimensionality reduction method(s).
Data split and cross-validation	Present the type of data split and the cross-validation method used. Depending on applicability, provide the size of the training set, test set, and validation set, as well as the number of folds. Include details such as whether stratification (stratified split) was used in splitting the dataset.
Algorithm/machine learning type	Specify the machine learning algorithm used (e.g., random forest, logistic regression).
Hyperparameters	List and range (options) of hyperparameters tuned for each algorithm. Present also the hyperparameter tuning method.
Performance metrics	List the evaluation metrics used for the algorithm(s), their values, and the formulas used to calculate the metric scores.
Normalization	Describe the scaling or normalization technique(s) applied, or if not required, explain why.
Missing values	Indicate if the data has missing values, the extent of missing data, and the methods used to address them.
Missingness mechanism	Report the category for the cause of the data to be missing: missing completely at random (MCAR), missing at random (MAR), or missing not at random (MNAR)
Resampling technique	Provide the name the resampling technique (if applicable).

***See* Table 1** ***for definitions of the technical terms used in this table.***

Given that most studies did not evaluate the missingness mechanism prior to imputation, that imputation may be based on assumptions that are not credible. We therefore suggest that authors describe the amount and structure of missingness, explore plausible mechanisms, and provide a rationale for the selected strategy. Several resources are available to assist authors and provide guidance on this matter [39,53–54].

With respect to metric selection, about a quarter of the included studies assessed anomaly detection performance using only one metric, with the vast majority relying on ROC AUC. We recommend against relying solely on ROC AUC, or any other single metric. ROC AUC does not indicate whether performance is acceptable at the operating thresholds used in practical settings. A high ROC AUC can still correspond to unacceptably high false-positive or false-negative rates. We therefore encourage authors to report additional threshold-specific measures at clinically meaningful cutoffs. Authors may also apply more analytic approaches, including decision curve analysis and net benefit, to quantify the clinical consequences of different threshold choices and the trade-off between false positives and false negatives.

### Limitations of the study

Our study has some limitations. Firstly, we only included peer-reviewed studies published in English. While non-peer-reviewed articles are often considered less credible, this criterion may have led to the exclusion of potentially credible and sound studies that could offer valuable insights. Additionally, by including only English-language publications, the applicability of our conclusions may be limited to studies published in English. Finally, our chosen search terms might not have captured all relevant studies. Some studies addressing medical anomaly detection may not explicitly use the key terms we used to target such studies, potentially resulting in the omission of relevant research.

## Conclusion

In conclusion, we conducted a scoping review of existing literature on medical anomaly detection based on routine healthcare data, highlighted current practices, and identified suboptimal practices that, if addressed properly, could enhance the reliability and reproducibility of future studies. Although the variety of machine learning algorithms and the growing number of studies signal a promising future for anomaly detection based on EHR data, several pitfalls in the implementation and reporting of these techniques impede their adequate use. We noted that more studies need to adhere to standard procedures while addressing the issue of missingness and be thorough in the utilization and reporting of performance metrics by selecting metrics that align with the study’s objectives, and by reporting the numbers of false positives and false negatives as well as discussing the preferred balance between them in the light of the clinical context of the study. Incorporating the consequences of false positives and false negatives is crucial as the usability of developed algorithms depends on their ability to support daily clinical tasks, which also have broader societal and economic impacts.

## Supporting information

S1 Text Description of the categories of unsupervised anomaly detection methods. (DOCX)

S1 Table PRISMA-ScR checklist. (DOCX)

S2 Table Search strategy. (DOCX)

S3 Table List of studies included in the scoping review. (DOCX)

S4 Table The number of included studies by country. (DOCX)

S5 Table Preprocessing actions reported in each included study. (DOCX)

S6 Table The characteristics of the datasets in the included studies. (DOCX)

S7 Table The metrics used to evaluate the anomaly detection algorithms (total count) by the included studies. (DOCX)

S8 Table The metrics used as sole performance indicators in the included studies. (DOCX)

S9 Table Summary of the categorical features (frequency) among the extracted features. (DOCX)

S10 Table List of machine learning algorithms used in the included articles. (DOCX)

S11 Table Summary for pairwise comparison between ML algorithms according to ROC AUC. (DOCX)

S12 Table Example of adjusting machine learning output to control false positives and negatives. (DOCX)
