# Supplementary material for: Detection of rare medical events in electronic health records using machine learning: Current practices and suggestions – A scoping review
Source: PLoS One. 2026 Mar 16;21(3):e0332963. doi: 10.1371/journal.pone.0332963 (PMC12991209; doi:10.1371/journal.pone.0332963)
Supplement: S1 Text — (DOCX) [file pone.0332963.s001.docx]

**S1 Text: Description of the categories of unsupervised anomaly detection methods**

*NN-based algorithms* are a category of algorithms based on the assumption that anomalous data points lie relatively far from their closest neighbors while normal data instances lie closer to each other. The quantification of how close the data points are to each other depends on the specific algorithm. E.g., kNN is based on the distance between a data point and its k-nearest neighbors, and the local outlier factor (LOF) is based on the density of data points around a certain data point relative to the density around its neighbors.

Existing *clustering algorithms* such as K-means and Density-Based Spatial Clustering of Applications with Noise (DBSCAN) are based on the assumption that anomalies either belong to a small cluster or do not belong to any cluster at all. Unlike NN-based methods, here anomalous points are identified based on their distance from formed clusters or based on the size of their cluster.

Recently, *subspace-based anomaly detection* has become a promising approach for finding anomalies in datasets with many variables, often exceeding the sample size in number. Subspace-based approaches emphasize on finding anomalous data points hidden in a subgroup of the full dataset, because anomalous data points could be undetectable when considering the entire dataset, but detectable when considering a selection (subspace) of variables.

*Statistical-based anomaly detection* is considered the oldest and most traditional anomaly detection approach. Statistical-based anomaly detection discriminates between anomalous and normal data points by computing measures that describe the statistical properties of the data such as mean, variance, and distribution. The statistical-based approach can be parametric (assumes a specific distribution for the data) or non-parametric (no assumption regarding the underlying distribution of the data). Statistical-based approaches usually serve as a baseline for other anomaly detection techniques.

*Classification-based:* identifying anomalous data points using a classification-based approach involves finding regions or boundaries for the normal data points. Then, any data points outside that boundary are classified as anomalous (one-class classification), such as one-class support vector machine (OCSVM) and one-class gaussian process (OCGP).

*Other*: algorithms that cannot be classified into any of the first five categories are placed in this category, which mainly encompasses methods that are an ensemble (combination) of two or more of the other categories.
