# Supplementary material for: Detection of rare medical events in electronic health records using machine learning: Current practices and suggestions – A scoping review
Source: PLoS One. 2026 Mar 16;21(3):e0332963. doi: 10.1371/journal.pone.0332963 (PMC12991209; doi:10.1371/journal.pone.0332963)
Supplement: S2 Table — (DOCX) [file pone.0332963.s003.docx]

**S2 Table: Search strategy**

| **Electronic database** | **Search query** | **Number of hits** |
| --- | --- | --- |
| **PubMed** | (("change point detection"[all fields] OR "outlier observation*"[all fields] OR "outlier detection"[all fields] OR "implausible value*"[all fields] OR "implausible observation*"[all fields] OR "anomaly detection"[all fields] OR "abnormality detection"[all fields] OR "novelty detection"[all fields]) AND (clinic*[all fields] OR Health[all fields] OR Healthcare[all fields] OR Patient[all fields] OR medical[all fields])) | **2114** |
| **Web of science** | ALL=(("change point detection" OR "outlier observation*" OR "outlier detection" OR "implausible value*" OR "implausible observation*" OR "anomaly detection" OR "abnormality detection" OR "novelty detection") AND (clinical OR health OR healthcare OR patient OR medical)) | **3884** |
