# Supplementary material for: Detection of rare medical events in electronic health records using machine learning: Current practices and suggestions – A scoping review
Source: PLoS One. 2026 Mar 16;21(3):e0332963. doi: 10.1371/journal.pone.0332963 (PMC12991209; doi:10.1371/journal.pone.0332963)
Supplement: S3 Table — (DOCX) [file pone.0332963.s004.docx]

**S3 Table: List of studies included in the scoping review**

| No. | References |
| --- | --- |
| 1 | A.G R, Abdulla MS, S A. Lightly trained support vector data description for novelty detection. Expert Systems with Applications. 2017;85:25-32. doi: https://doi.org/10.1016/j.eswa.2017.05.007. |
| 2 | Abdoh SF, Rizka MA, Maghraby FA. Cervical cancer diagnosis using random forest classifier with SMOTE and feature reduction techniques. IEEE Access. 2018;6:59475-85. |
| 3 | Abdualgalil B, Abraham S, Ismael WM. Covid-19 infection prediction using efficient machine learning techniques based on clinical data. Journal of Advances in Information Technology. 2022;13(5). |
| 4 | Adem K, Kılıçarslan S. COVID-19 Diagnosis Prediction in Emergency Care Patients using Convolutional Neural Network. Afyon Kocatepe Üniversitesi Fen ve Mühendislik Bilimleri Dergisi. 2021;21(2):300-9. doi: 10.35414/akufemubid.788898. |
| 5 | Akhtar A, Abbas N. Prediction of COVID-19 using ensemble based machine learning approach. International Journal of Computational and Innovative Sciences. 2022;1(4):34-41. |
| 6 | Aljameel SS, Khan IU, Aslam N, Aljabri M, Alsulmi ES. Machine Learning‐Based Model to Predict the Disease Severity and Outcome in COVID‐19 Patients. Scientific programming. 2021;2021(1):5587188. |
| 7 | Anderson JP, Parikh JR, Shenfeld DK, Ivanov V, Marks C, Church BW, et al. Reverse Engineering and Evaluation of Prediction Models for Progression to Type 2 Diabetes: An Application of Machine Learning Using Electronic Health Records. (1932-2968 (Electronic)). |
| 8 | Azari A, Janeja VP, Levin S, editors. Imbalanced learning to predict long stay Emergency Department patients. 2015 IEEE International Conference on Bioinformatics and Biomedicine (BIBM); 2015 9-12 Nov. 2015. |
| 9 | Banerjee A, Ray S, Vorselaars B, Kitson J, Mamalakis M, Weeks S, et al. Use of Machine Learning and Artificial Intelligence to predict SARS-CoV-2 infection from Full Blood Counts in a population. (1878-1705 (Electronic)). |
| 10 | Batsakis S, Adamou M, Tachmazidis I, Antoniou G, Kehagias T, editors. Data-driven decision support for autism diagnosis using machine learning2021. |
| 11 | Bernardini M, Romeo L, Misericordia P, Frontoni E. Discovering the Type 2 Diabetes in Electronic Health Records Using the Sparse Balanced Support Vector Machine. IEEE Journal of Biomedical and Health Informatics. 2020;24(1):235-46. doi: 10.1109/JBHI.2019.2899218. |
| 12 | Bhattacharya M, Jurkovitz C, Shatkay H. Chronic Kidney Disease stratification using office visit records: Handling data imbalance via hierarchical meta-classification. BMC Medical Informatics and Decision Making. 2018;18:35-44. |
| 13 | Chadaga K, Prabhu S, Bhat KV, Umakanth S, Sampathila N. Medical Diagnosis of COVID-19 Using Blood Tests and Machine Learning. Journal of Physics: Conference Series. 2022;2161(1):012017. doi: 10.1088/1742-6596/2161/1/012017. |
| 14 | Chan FTS, Wang ZX, Patnaik S, Tiwari MK, Wang XP, Ruan JH. Ensemble-learning based neural networks for novelty detection in multi-class systems. Applied Soft Computing. 2020;93:106396. |
| 15 | Chicco D, Lovejoy CA, Oneto L. A machine learning analysis of health records of patients with chronic kidney disease at risk of cardiovascular disease. IEEE Access. 2021;9:165132-44. |
| 16 | Cho BH, Yu H, Kim K-W, Kim TH, Kim IY, Kim SI. Application of irregular and unbalanced data to predict diabetic nephropathy using visualization and feature selection methods. Artificial intelligence in medicine. 2008;42(1):37-53. |
| 17 | Choi DH, Park JH, Choi YH, Song KJ, Kim S, Shin SD. Machine learning analysis to identify data entry errors in prehospital patient care reports: a case study of a national out-of-hospital cardiac arrest registry. Prehospital Emergency Care. 2024;28(1):14-22. |
| 18 | Churpek MM, Yuen TC, Winslow C, Meltzer DO, Kattan MW, Edelson DP. Multicenter comparison of machine learning methods and conventional regression for predicting clinical deterioration on the wards. Critical care medicine. 2016;44(2):368-74. |
| 19 | Clifton DA, Clifton L, Hugueny S, Wong D, Tarassenko L. An extreme function theory for novelty detection. IEEE Journal of Selected Topics in Signal Processing. 2012;7(1):28-37. |
| 20 | Cohen G, Hilario M, Sax H, Hugonnet S, Pellegrini C, Geissbuhler A, editors. An application of one-class support vector machines to nosocomial infection detection2004: IOS press. |
| 21 | Dagliati A, Marini S, Sacchi L, Cogni G, Teliti M, Tibollo V, et al. Machine learning methods to predict diabetes complications. Journal of diabetes science and technology. 2018;12(2):295-302. |
| 22 | Dairi A, Harrou F, Sun Y. Deep generative learning-based 1-svm detectors for unsupervised covid-19 infection detection using blood tests. IEEE Transactions on Instrumentation and Measurement. 2021;71:1-11. |
| 23 | Davoudi A, Ozrazgat-Baslanti T, Ebadi A, Bursian AC, Bihorac A, Rashidi P, editors. Delirium prediction using machine learning models on predictive electronic health records data2017: IEEE. |
| 24 | de Freitas Barbosa VA, Gomes JC, de Santana MA, Albuquerque JEdA, de Souza RG, de Souza RE, et al. Heg. IA: an intelligent system to support diagnosis of Covid-19 based on blood tests. Research on Biomedical Engineering. 2021:1-18. |
| 25 | Dezman Zd Fau - Gao C, Gao C Fau - Yang S, Yang S Fau - Hu P, Hu P Fau - Yao L, Yao L Fau - Li H-C, Li Hc Fau - Chang C-I, et al. Anomaly Detection Outperforms Logistic Regression in Predicting Outcomes in Trauma Patients. (1545-0066 (Electronic)). |
| 26 | Dingle K, Zimek A, Azizieh F, Ansari AR. Establishing a many-cytokine signature via multivariate anomaly detection. Scientific Reports. 2019;9(1):9684. |
| 27 | Dogan A, Birant D. A Two-Level Approach based on Integration of Bagging and Voting for Outlier Detection. Journal of Data and Information Science. 2020;5(2):111-35. doi: doi:10.2478/jdis-2020-0014. |
| 28 | Du H, Zhao S, Zhang D, Wu J, editors. Novel clustering-based approach for local outlier detection2016: IEEE. |
| 29 | Elmogy A, Rizk H, Sarhan AM. Ofcod: On the fly clustering based outlier detection framework. Data. 2020;6(1):1. |
| 30 | Fan R, Zhang N, Yang L, Ke J, Zhao D, Cui Q. AI-based prediction for the risk of coronary heart disease among patients with type 2 diabetes mellitus. Scientific reports. 2020;10(1):14457. |
| 31 | Fitriyani NL, Syafrudin M, Alfian G, Rhee J. Development of disease prediction model based on ensemble learning approach for diabetes and hypertension. Ieee Access. 2019;7:144777-89. |
| 32 | Geetha R, Sivasubramanian S, Kaliappan M, Vimal S, Annamalai S. Cervical cancer identification with synthetic minority oversampling technique and PCA analysis using random forest classifier. Journal of medical systems. 2019;43:1-19. |
| 33 | Goldstein M, Uchida S. A Comparative Evaluation of Unsupervised Anomaly Detection Algorithms for Multivariate Data. PLOS ONE. 2016;11(4):e0152173. doi: 10.1371/journal.pone.0152173. |
| 34 | Han L, Luo S, Yu J, Pan L, Chen S. Rule extraction from support vector machines using ensemble learning approach: an application for diagnosis of diabetes. IEEE journal of biomedical and health informatics. 2014;19(2):728-34. |
| 35 | Hao B, Hu Y, Adams WG, Assoumou SA, Hsu HE, Bhadelia N, et al. A GPT-based EHR modeling system for unsupervised novel disease detection. Journal of Biomedical Informatics. 2024;157:104706. |
| 36 | Hauskrecht M, Valko M Fau - Batal I, Batal I Fau - Clermont G, Clermont G Fau - Visweswaran S, Visweswaran S Fau - Cooper GF, Cooper GF. Conditional outlier detection for clinical alerting. (1942-597X (Electronic)). |
| 37 | Hauskrecht M, Valko M, Kveton B, Visweswaran S, Cooper GF, editors. Evidence-based anomaly detection in clinical domains2007: American Medical Informatics Association. |
| 38 | He Z, Xu X, Deng S. Discovering cluster-based local outliers. Pattern recognition letters. 2003;24(9-10):1641-50. |
| 39 | Ho KC, Speier W, El-Saden S, Liebeskind DS, Saver JL, Bui AAT, et al. Predicting Discharge Mortality after Acute Ischemic Stroke Using Balanced Data. AMIA Annual Symposium Proceedings. 2014;2014:1787-96. |
| 40 | Hu C, Anjur V, Saboo K, Reddy KR, O'Leary J, Tandon P, et al. Low predictability of readmissions and death using machine learning in cirrhosis. Official journal of the American College of Gastroenterology\| ACG. 2021;116(2):336-46. |
| 41 | Huang Y, McCullagh P, Black N, Harper R. Feature selection and classification model construction on type 2 diabetic patients’ data. Artificial intelligence in medicine. 2007;41(3):251-62. |
| 42 | Huang Z, Dong W, Ji L, Yin L, Duan H. On local anomaly detection and analysis for clinical pathways. Artificial intelligence in medicine. 2015;65(3):167-77. |
| 43 | Huang Z, Lu X, Duan H. Anomaly detection in clinical processes. AMIA Annu Symp Proc. 2012;2012:370-9. Epub 2012/11/03. PubMed PMID: 23304307. |
| 44 | Ibrahim OA, Fu S, Vassilaki M, Mielke MM, St Sauver J, Petersen RC, et al., editors. Detection of dementia signals from longitudinal clinical visits using one-class classification2022: IEEE. |
| 45 | Ijaz MF, Alfian G, Syafrudin M, Rhee J. Hybrid prediction model for type 2 diabetes and hypertension using DBSCAN-based outlier detection, synthetic minority over sampling technique (SMOTE), and random forest. Applied Sciences. 2018;8(8):1325. |
| 46 | Ijaz MF, Attique M, Son Y. Data-Driven Cervical Cancer Prediction Model with Outlier Detection and Over-Sampling Methods. Sensors [Internet]. 2020; 20(10). |
| 47 | Jahangir M, Afzal H, Ahmed M, Khurshid K, Amjad MF, Nawaz R, et al. Auto-MeDiSine: an auto-tunable medical decision support engine using an automated class outlier detection method and AutoMLP. Neural Computing and Applications. 2020;32:2621-33. |
| 48 | Jian Y, Pasquier M, Sagahyroon A, Aloul F, editors. A machine learning approach to predicting diabetes complications2021: MDPI. |
| 49 | Jiang F, Chen Y-M. Outlier detection based on granular computing and rough set theory. Applied Intelligence. 2015;42(2):303-22. doi: 10.1007/s10489-014-0591-4. |
| 50 | Jiang F, Sui Y, Cao C. A hybrid approach to outlier detection based on boundary region. Pattern recognition letters. 2011;32(14):1860-70. |
| 51 | Jiang S-y, An Q-b, editors. Clustering-based outlier detection method2008: IEEE. |
| 52 | Joshi RP, Pejaver V, Hammarlund NE, Sung H, Lee SK, Furmanchuk Ao, et al. A predictive tool for identification of SARS-CoV-2 PCR-negative emergency department patients using routine test results. Journal of Clinical Virology. 2020;129:104502. |
| 53 | Kara A. Accurate detection of coronavirus cases using deep learning with attention mechanism and genetic algorithm. Multimedia Tools and Applications. 2024;83(34):81477-90. |
| 54 | Karajizadeh M, Nasiri M, Yadollahi M, Zolfaghari AH, Pakdam A. Mortality prediction from hospital-acquired infections in trauma patients using an unbalanced dataset. Healthcare informatics research. 2020;26(4):284-94. |
| 55 | Khan W, Zaki N, Ahmad A, Masud MM, Govender R, Rojas-Perilla N, et al. Node embedding-based graph autoencoder outlier detection for adverse pregnancy outcomes. Scientific reports. 2023;13(1):19817. |
| 56 | Khatibi T, Kheyrikoochaksarayee N, Sepehri MM. Analysis of big data for prediction of provider-initiated preterm birth and spontaneous premature deliveries and ranking the predictive features. Archives of gynecology and obstetrics. 2019;300:1565-82. |
| 57 | Koko RRZ, Yassine IA, Wahed MA, Madete JK, Rushdi MA. Dynamic construction of outlier detector ensembles with bisecting k-means clustering. IEEE Access. 2023;11:24431-47. |
| 58 | Krishnamurthy S, Ks K, Dovgan E, Luštrek M, Gradišek Piletič B, Srinivasan K, et al., editors. Machine learning prediction models for chronic kidney disease using national health insurance claim data in Taiwan2021: MDPI. |
| 59 | Kukar M, Gunčar G, Vovko T, Podnar S, Černelč P, Brvar M, et al. COVID-19 diagnosis by routine blood tests using machine learning. Scientific reports. 2021;11(1):10738. |
| 60 | Lancia G, Varkila MRJ, Cremer OL, Spitoni C. Two-step interpretable modeling of ICU-AIs. Artificial Intelligence in Medicine. 2024;151:102862. |
| 61 | Lazarevic A, Kumar V. Feature bagging for outlier detection2005. 157-66 p. |
| 62 | Li J, Li J, Wang C, Verbeek FJ, Schultz T, Liu H. MS2OD: outlier detection using minimum spanning tree and medoid selection. Machine Learning: Science and Technology. 2024;5(1):015025. |
| 63 | Li J, Li J, Wang C, Verbeek FJ, Schultz T, Liu H. Outlier detection using iterative adaptive mini-minimum spanning tree generation with applications on medical data. Frontiers in Physiology. 2023;14:1233341. |
| 64 | Li J, Liu L, Sun J, Mo H, Yang J-J, Chen S, et al. Comparison of different machine learning approaches to predict small for gestational age infants. IEEE Transactions on Big Data. 2016;6(2):334-46. |
| 65 | Lin CH, Hsu KC, Johnson KR, Luby M, Fann YC. Applying density-based outlier identifications using multiple datasets for validation of stroke clinical outcomes. (1872-8243 (Electronic)). |
| 66 | Liu L, Ni Y, Zhang N, Nick Pratap J. Mining patient-specific and contextual data with machine learning technologies to predict cancellation of children's surgery. Int J Med Inform. 2019;129:234-41. Epub 20190608. doi: 10.1016/j.ijmedinf.2019.06.007. PubMed PMID: 31445261. |
| 67 | Liu L, Wu DTY, Spooner SA, Ni Y, editors. Development and evaluation of an automated approach to detect weight abnormalities in pediatric weight charts2021: American Medical Informatics Association. |
| 68 | Luca SE, Pimentel MAF, Watkinson PJ, Clifton DA. Point process models for novelty detection on spatial point patterns and their extremes. Computational Statistics & Data Analysis. 2018;125:86-103. |
| 69 | Luo Y, Li Z, Guo H, Cao H, Song C, Guo X, et al. Predicting congenital heart defects: A comparison of three data mining methods. PloS one. 2017;12(5):e0177811. |
| 70 | Luo Y, Szolovits P, Dighe AS, Baron JM. Using Machine Learning to Predict Laboratory Test Results. (1943-7722 (Electronic)). |
| 71 | Marimuthu P, Perumal V, Vijayakumar V. Intelligent personalized abnormality detection for remote health monitoring. International Journal of Intelligent Information Technologies (IJIIT). 2020;16(2):87-109. |
| 72 | Mayanglambam SD, Horng S-J, Pamula R. PSO clustering and pruning-based KNN for outlier detection. Soft Computing. 2023:1-17. |
| 73 | Mi H, editor Discovering local outlier based on rough clustering2011: IEEE. |
| 74 | Mienye ID, Sun Y. Performance analysis of cost-sensitive learning methods with application to imbalanced medical data. Informatics in Medicine Unlocked. 2021;25:100690. |
| 75 | Mohammed AJ, Muhammed Hassan M, Hussein Kadir D. Improving classification performance for a novel imbalanced medical dataset using SMOTE method. International Journal of Advanced Trends in Computer Science and Engineering. 2020;9(3):3161-72. |
| 76 | Mohammedqasim H, Biabani SAA, Ata O, Alomary MN, Almehmadi M, Alsairi AA, et al. Multi-objective deep learning framework for COVID-19 dataset problems. Journal of King Saud University-Science. 2023;35(3):102527. |
| 77 | Murphree D, Ngufor C, Upadhyaya S, Madde N, Clifford L, Kor DJ, et al., editors. Ensemble learning approaches to predicting complications of blood transfusion2015: IEEE. |
| 78 | Nagata K, Tsuji T, Suetsugu K, Muraoka K, Watanabe H, Kanaya A, et al. Detection of overdose and underdose prescriptions—An unsupervised machine learning approach. PloS one. 2021;16(11):e0260315. |
| 79 | Nathaniel SFPS, Alwarsamy K, Viswanathan R, Subramanian GV, Veerabahu V. The influence of feature grouping algorithm in outlier detection with categorical data. Acta Scientiarum Technology. 2024;46:e66902. |
| 80 | Niu H, Omitaomu OA, Langston MA, Olama M, Ozmen O, Klasky HB, et al. EHR-BERT: A BERT-based model for effective anomaly detection in electronic health records. Journal of Biomedical Informatics. 2024;150:104605. |
| 81 | Noor S, Siddiqui A, Saleem MI, Minhas HN. Using Machine Learning for Identifying COVID-19. JOURNAL OF INFORMATION & COMMUNICATION TECHNOLOGY. 2023;17(1):1-7. |
| 82 | Perveen S, Shahbaz M, Guergachi A, Keshavjee K. Performance analysis of data mining classification techniques to predict diabetes. Procedia Computer Science. 2016;82:115-21. |
| 83 | Pratap V, Singh AP. A Novel Clustering-Based Three Level Under-Sampling Algorithm For Class Imbalance Problem. Journal of Applied Science and Engineering. 2024;27(4):2389-99. |
| 84 | Qiu H, Yu H-Y, Wang L-Y, Yao Q, Wu S-N, Yin C, et al. Electronic health record driven prediction for gestational diabetes mellitus in early pregnancy. Scientific reports. 2017;7(1):16417. |
| 85 | Ren H, Li Y, Huang T. Anomaly Detection models for SARS-CoV-2 Surveillance based on genome k-mers. Microorganisms. 2023;11(11):2773. |
| 86 | Ren K, Yang H, Zhao Y, Chen W, Xue M, Miao H, et al. A Robust AUC Maximization Framework With Simultaneous Outlier Detection and Feature Selection for Positive-Unlabeled Classification. IEEE Transactions on Neural Networks and Learning Systems. 2019;30(10):3072-83. doi: 10.1109/TNNLS.2018.2870666. |
| 87 | Rubini PE, Subasini CA, Katharine AV, Kumaresan V, Kumar SG, Nithya TM. A cardiovascular disease prediction using machine learning algorithms. Annals of the Romanian Society for Cell Biology. 2021;25(2):904-12. |
| 88 | Samariya D, Ma J, Aryal S, Zhao X. Detection and explanation of anomalies in healthcare data. Health Information Science and Systems. 2023;11(1):20. |
| 89 | Santiso S, Casillas A, Pérez A. The class imbalance problem detecting adverse drug reactions in electronic health records. Health informatics journal. 2019;25(4):1768-78. |
| 90 | Santos HDPd, Ulbrich AHDPS, Woloszyn V, Vieira R. DDC-Outlier: Preventing Medication Errors Using Unsupervised Learning. IEEE Journal of Biomedical and Health Informatics. 2019;23(2):874-81. doi: 10.1109/JBHI.2018.2828028. |
| 91 | Scrutinio D, Ricciardi C, Donisi L, Losavio E, Battista P, Guida P, et al. Machine learning to predict mortality after rehabilitation among patients with severe stroke. Scientific reports. 2020;10(1):20127. |
| 92 | Shackelford S, Yang S, Hu P, Miller C, Anazodo A, Galvagno S, et al. Predicting blood transfusion using automated analysis of pulse oximetry signals and laboratory values. Journal of Trauma and Acute Care Surgery. 2015;79(4):S175-S80. |
| 93 | Sugiyama M, Borgwardt K. Rapid distance-based outlier detection via sampling. Advances in neural information processing systems. 2013;26. |
| 94 | Syed Z, Rubinfeld I, editors. Unsupervised risk stratification in clinical datasets: Identifying patients at risk of rare outcomes2010: Citeseer. |
| 95 | Syed Z, Saeed M, Rubinfeld I, editors. Identifying high-risk patients without labeled training data: anomaly detection methodologies to predict adverse outcomes2010: American Medical Informatics Association. |
| 96 | Symum HA-OX, Zayas-Castro JA-OX. Prediction of Chronic Disease-Related Inpatient Prolonged Length of Stay Using Machine Learning Algorithms. (2093-3681 (Print)). |
| 97 | Tang B, He H. A local density-based approach for outlier detection. Neurocomputing. 2017;241:171-80. doi: https://doi.org/10.1016/j.neucom.2017.02.039. |
| 98 | Tong A, Wolf G, Krishnaswamyt S, editors. Fixing bias in reconstruction-based anomaly detection with lipschitz discriminators2020: IEEE. |
| 99 | Wang J, Jia Y, Wang D, Xiao W, Wang Z. Weighted IForest and siamese GRU on small sample anomaly detection in healthcare. Computer Methods and Programs in Biomedicine. 2022;218:106706. |
| 100 | Wang K, Tian J, Zheng C, Yang H, Ren J, Li C, et al. Improving risk identification of adverse outcomes in chronic heart failure using SMOTE+ ENN and machine learning. Risk management and healthcare policy. 2021:2453-63. |
| 101 | Wang L, Zhang Q, Niu X, Ren Y, Xia J. Outlier Detection of Mixed Data Based on Neighborhood Combinatorial Entropy. Computers, Materials and Continua. 2021;69(2):1765-81. doi: https://doi.org/10.32604/cmc.2021.017516. |
| 102 | Xiong Z-Y, Long H, Zhang Y-F, Wang X-X, Gao Q-Q, Li L-T, et al. A neighborhood weighted-based method for the detection of outliers. Applied Intelligence. 2023;53(9):9897-915. |
| 103 | Xu X, Liu H, Li L, Yao M. A comparison of outlier detection techniques for high-dimensional data. International Journal of Computational Intelligence Systems. 2018;11(1):652. |
| 104 | Yahyaoui A, Rasheed J, Alsubai S, Shubair RM, Alqahtani A, İşler B, et al. Performance comparison of deep and machine learning approaches toward COVID-19 detection. Intelligent Automation and Soft Computing. 2023. |
| 105 | Yan K, You X, Ji X, Yin G, Yang F, editors. A Hybrid Outlier Detection Method for Health Care Big Data. 2016 IEEE International Conferences on Big Data and Cloud Computing (BDCloud), Social Computing and Networking (SocialCom), Sustainable Computing and Communications (SustainCom) (BDCloud-SocialCom-SustainCom); 2016 8-10 Oct. 2016. |
| 106 | Yang F, Soriano J, Kubo T, Ikeda K, editors. Application of SsVGMM to medical data-classification with novelty detection2017: IEEE. |
| 107 | Yang H, Li X, Cao H, Cui Y, Luo Y, Liu J, et al. Using machine learning methods to predict hepatic encephalopathy in cirrhotic patients with unbalanced data. Computer methods and programs in biomedicine. 2021;211:106420. |
| 108 | Yang X, Zhuang Y, Shi M, Cao X, Chen D, Tang Y. SPiForest: an anomaly detecting algorithm using space partition constructed by probability density-based inverse sampling. IEEE Transactions on Neural Networks and Learning Systems. 2022. |
| 109 | Yang Y, Fan C, Chen L, Xiong H. IPMOD: An efficient outlier detection model for high-dimensional medical data streams. Expert Systems with Applications. 2022;191:116212. |
| 110 | Yousef H, Feng SF, Jelinek HF. Exploratory risk prediction of type II diabetes with isolation forests and novel biomarkers. Scientific Reports. 2024;14(1):14409. |
| 111 | Yuan Z, Chen B, Liu J, Chen H, Peng D, Li P. Anomaly detection based on weighted fuzzy-rough density. Applied Soft Computing. 2023;134:109995. |
| 112 | Zarkogianni K, Athanasiou M, Thanopoulou AC, Nikita KS. Comparison of machine learning approaches toward assessing the risk of developing cardiovascular disease as a long-term diabetes complication. IEEE journal of biomedical and health informatics. 2017;22(5):1637-47. |
| 113 | Zhang CK, Yin A, Zuo W, Chen YY. Privacy preserving anomaly detection based on local density estimation. (1551-0018 (Electronic)). |
| 114 | Zhang J, Li Z, Nai K, Gu Y, Sallam A. DELR: A double-level ensemble learning method for unsupervised anomaly detection. Knowledge-Based Systems. 2019;181:104783. |
| 115 | Zhang L, Wang Y, Niu M, Wang C, Wang Z. Nonlaboratory-based risk assessment model for type 2 diabetes mellitus screening in Chinese rural population: a joint bagging-boosting model. IEEE Journal of Biomedical and Health Informatics. 2021;25(10):4005-16. |
| 116 | Zhang Z, Hou Y, Liu D, Zhang R, Guo X. HGOD: Outlier detection based on a hybrid graph. Neurocomputing. 2024;602:128288. |
| 117 | Zheng J, Li J, Liu C, Wang J, Li J, Liu H. Anomaly detection for high-dimensional space using deep hypersphere fused with probability approach. Complex & Intelligent Systems. 2022;8(5):4205-20. |
