# Supplementary material for: Detection of rare medical events in electronic health records using machine learning: Current practices and suggestions – A scoping review
Source: PLoS One. 2026 Mar 16;21(3):e0332963. doi: 10.1371/journal.pone.0332963 (PMC12991209; doi:10.1371/journal.pone.0332963)
Supplement: S4 Table — (DOCX) [file pone.0332963.s005.docx]

**S4 Table: The number of included studies by country**

| **Country** | **No. of publication** |
| --- | --- |
| USA | 44 |
| Brazil | 13 |
| China | 12 |
| Italy | 4 |
| UK | 4 |
| Venezuela | 3 |
| South Korea | 3 |
| UAE | 2 |
| India | 2 |
| Australia | 2 |
| Iran | 2 |
| Canada | 2 |
| Taiwan | 2 |
| Türkiye | 1 |
| Indonesia | 1 |
| Saudi Arabia | 1 |
| Spain | 1 |
| Slovenia | 1 |
| Switzerland | 1 |
| Iraq | 1 |
| Greece | 1 |
| Netherlands | 1 |
| Argentina | 1 |
