# Supplementary material for: Detection of rare medical events in electronic health records using machine learning: Current practices and suggestions – A scoping review
Source: PLoS One. 2026 Mar 16;21(3):e0332963. doi: 10.1371/journal.pone.0332963 (PMC12991209; doi:10.1371/journal.pone.0332963)
Supplement: S5 Table — (DOCX) [file pone.0332963.s006.docx]

**S5 Table: Preprocessing actions reported in each included study**

| **Study** | **Data split** | **Outlier detection** | **Normalization**  **or scaling** | **Missing data handling** | **Feature selection** |
| --- | --- | --- | --- | --- | --- |
| 1 | X |  |  |  |  |
| 2 | X |  |  | X |  |
| 3 | X |  | X |  | X |
| 4 | X |  | X | X |  |
| 5 | X |  | X |  | X |
| 6 | X |  |  | X | X |
| 7 | X |  |  | X |  |
| 8 | X |  |  | X | X |
| 9 | X |  | X | X |  |
| 10 | X |  | X | X |  |
| 11 | X |  |  |  |  |
| 12 | X |  |  | X |  |
| 13 | X |  | X | X | X |
| 14 | X |  |  | X |  |
| 15 | X |  |  | X |  |
| 16 | X |  |  |  |  |
| 17 | X | X | X | X | X |
| 18 | X |  |  | X |  |
| 19 | X |  | X | X |  |
| 20 | X |  |  | X | X |
| 21 | X |  |  | X | X |
| 22 | X |  | X | X | X |
| 23 | X | X | X | X | X |
| 24 | X |  | X | X | X |
| 25 | X |  |  |  | X |
| 26 |  |  | X | X |  |
| 27 |  |  |  |  | X |
| 28 |  |  |  |  |  |
| 29 | X |  | X |  |  |
| 30 |  |  |  |  | X |
| 31 | X |  |  | X |  |
| 32 | X |  |  | X | X |
| 33 |  |  |  |  |  |
| 34 | X | X | X | X | X |
| 35 | X |  | X |  | X |
| 36 | X |  |  |  | X |
| 37 | X |  |  |  | X |
| 38 |  |  |  |  |  |
| 39 | X |  |  | X | X |
| 40 | X |  |  | X | X |
| 41 | X | X |  | X | X |
| 42 |  |  |  |  |  |
| 43 | X |  |  |  |  |
| 44 | X |  |  | X |  |
| 45 | X |  | X | X | X |
| 46 | X | X |  | X | X |
| 47 | X |  |  |  |  |
| 48 | X |  | X | X | X |
| 49 |  |  |  |  |  |
| 50 |  |  |  |  | X |
| 51 |  |  |  |  |  |
| 52 | X |  |  |  | X |
| 53 | X |  | X | X | X |
| 54 | X | X |  | X |  |
| 55 |  |  |  |  | X |
| 56 | X |  | X | X | X |
| 57 |  |  |  |  |  |
| 58 | X |  |  | X |  |
| 59 | X |  | X | X | X |
| 60 |  |  |  | X | X |
| 61 | X |  | X |  |  |
| 62 |  |  |  |  |  |
| 63 |  |  | X |  |  |
| 64 | X |  |  | X | X |
| 65 | X | X |  |  | X |
| 66 | X |  |  | X | X |
| 67 | X |  | X | X |  |
| 68 | X |  |  |  |  |
| 69 | X |  |  |  | X |
| 70 | X |  |  | X | X |
| 71 | X | X | X |  |  |
| 72 |  |  |  |  |  |
| 73 |  |  |  |  |  |
| 74 | X |  | X | X | X |
| 75 | X |  |  |  |  |
| 76 | X |  |  | X | X |
| 77 | X |  |  | X | X |
| 78 | X | X | X |  |  |
| 79 |  |  | X |  |  |
| 80 | X |  |  |  |  |
| 81 | X |  | X | X | X |
| 82 | X |  |  |  | X |
| 83 | X |  |  |  |  |
| 84 | X |  | X | X | X |
| 85 | X |  |  | X |  |
| 86 | X |  |  |  | X |
| 87 | X |  |  | X |  |
| 88 |  |  |  |  |  |
| 89 | X |  |  |  |  |
| 90 |  | X |  |  | X |
| 91 | X |  |  |  | X |
| 92 | X |  |  | X | X |
| 93 |  |  | X | X | X |
| 94 | X |  | X |  | X |
| 95 |  |  |  | X |  |
| 96 | X | X |  | X | X |
| 97 |  |  | X |  |  |
| 98 | X |  | X |  |  |
| 99 |  |  |  |  |  |
| 100 | X |  | X | X | X |
| 101 |  |  | X |  |  |
| 102 |  |  | X | X |  |
| 103 |  |  |  |  |  |
| 104 | X |  | X |  | X |
| 105 |  |  |  |  | X |
| 106 | X |  | X |  |  |
| 107 | X |  |  | X | X |
| 108 |  |  |  |  |  |
| 109 |  |  |  |  | X |
| 110 | X |  | X |  | X |
| 111 |  |  | X | X |  |
| 112 | X |  | X |  |  |
| 113 |  |  | X |  |  |
| 114 | X |  | X | X | X |
| 115 | X | X | X | X |  |
| 116 |  |  |  |  |  |
| 117 | X |  |  |  |  |

*The table summarizes whether the preprocessing actions (data split, outlier detection, normalization/scaling, missing data handling, and feature selection) were reported in the included studies. See S3 Table for the list of studies.*
