# Supplementary material for: Detection of rare medical events in electronic health records using machine learning: Current practices and suggestions – A scoping review
Source: PLoS One. 2026 Mar 16;21(3):e0332963. doi: 10.1371/journal.pone.0332963 (PMC12991209; doi:10.1371/journal.pone.0332963)
Supplement: S6 Table — (DOCX) [file pone.0332963.s007.docx]

**S6 Table: List of machine learning algorithms used in the included articles**

| **ML algorithm type** | **Varieties** |
| --- | --- |
| Supervised | AdaBoost, Bagging, Bagged trees, boosted C5.0, EBNN, ESOM, GBL, GBM, JBM, LightGBM, RF, Stacking, XGBoost, LS-SVM, SCV-balanced, SVM(Gaussian), SVM(Linear), SVM(Polynomial E1), WSVM, SVM(Polynomial E2), SVM (Polynomial E3), SVM (RBF) ,CART, CHAID, C4.5, C5.0, LR(Lasso), LR(Ridge), LR (spline), SLR, BiLSTM, DBN, FNNs, HWNN, LSTM, MLP, Perceptron, RBF, RBFN, RNNs, NB, kNN, BN, NS, BBHA, NBTree, One Rule, K-star, XGBOD, Regression based abnormality detection, DT, CNN, BBN, GAM, LDA, SOMs, Diverse ML-based ensemble learning |
| Unsupervised | ABOD, ABOF, aLOCI, CBOF, COF, DIS, GLOSH, INFLO, LOCI, LOF, LOF-UB, LoOP, MNN, ODIN, PB-KNN, RDOS, BIRCH, CBLOF, CMGOS , DBSCAN, FindCBLOF, HClust, LDCOF, OPTICS, RMFB, SClust, uCBLOF, ARMA, EFT, GUMM, HBOS, HMM, KDE, KDEOS, KLD, kNN(unsupervised), LDEM, MCD, PPM, SGP, Boundary and Distance(BD), Granular Computing(GrC), COP, AETD-OCC, HiCS, MVE, Node2Vec, WATCH, Softmax, Temporal pairwise similarity, iForest, ALOCC, DSVDD, Breadth first approach, DeepLog, ECOD,EHER-BERT, Edit distance, K-mean, LogCluster, LogAnomaly, qSp, kth-NN, kNN-DTW, kmeansTD, iNNE, WIF-SGRU, WFRDA, SUOD, SPiForest, SVDD, SOD, SGTD, Subsampling based anomaly detection, Boundary based anomaly detection, TOD, RMF, PSO, OFCOD, ODGrCR, OCSVM, OCGP, NaNOD, NWOD, NLOD, MUSC, RDOF, KRXD AD, CE, HDBSCAN, CARE, CBDS, CBOD, COPOD, CPM, FRP, CumSum, autoencoder, DAE, DB, DCROD, DDC, DELR, FBOD, EWS, IPMOD, RC, FB, FG, GAE, GMM, GPT, HGOD, INS, WDOD, KNFST, LAD, LDOF, LODA, LSCP, LUNAR, LogBERT, MEB, MGTD, MMOD, MOGAAL, MS2OD, RCLOS |
| Semi-supervised | SsVGMM, BAUC, ERR |

*ABOF, angle-based outlier factor; ABOD, angle based outlier detection; AETD, deep learning-based one class classifier; ALOCI, approximate local correlation integral; ARMA, auto regressive moving average; BAUC, blind area under the curve; BBN, Bayesian belief network; BiLSTM, Bidirectional long short term memory networks; BIRCH, balanced iterative reducing and clustering using hierarchies; Boundary based anomaly detection; BV-LOF, Bagged and Voted Local Outlier Detection; CARE, cumulative agreement rates ensemble; CART, Classification And Regression Tree; CBDS, clustering-based dynamic selection; CBLOF, cluster based local outlier factor; CBOD, Clustering-based outlier detection method; CBOF, connectivity based outlier factor; CE, Cluster based estimation; CHAID, Chi-square automatic interaction detection Tree; Children’s Hospital of Philadelphia; CMGOS, clustering based multivariate gaussian outlier score; CNN, convolutional neural network; CNN-LSTM, convolutional neural network - long short term memory; COF, connectivity based outlier factor; COP, Correlation Outlier Probabilities; COPOD, Copula based outlier detection; CPM, clinical pathway model; CumSum, cumulative sum approach; DAE, denoising autoencoder; DB, density based algorithm; DBN, Deep Belief Network; DBSCAN, density-based spatial clustering of applications with noise; DCROD, directed density ratio changing rate-based outlier detection; DDC, density distance centrality; DELR, double level ensemble learning;
DNN, Deep Neural Network; DT, decision tree; EBNN, Ensemble based learning of neural networks;
EFT, Extreme Function Theory; ERR, Error Minimization Model; ESOM, Ensemble Self Organizing Maps; EWS, early warning score; FB, feature bagging; FBOD, fluctuation-based outlier detection;
FG, feature grouping based unsupervised algorithm; FindCBLOF, Finding Clusters and Outliers based on Local Outlier Factor; FNNs, Forward neural networks; FRP, fast random projection + Node2Vec;
GAM, generalized additive model; GAE, graph autoencoder (GAE); GBM, Gradient boosting machine; GLOSH, Global-Local Outlier Scores from Hierarchies; GMM, gaussian mixture model;
GPT, generative pre-trained transformer; GUMM, Gaussian Uniform Mixture Model; HBOS, Histogram-based Outlier Score; HClust, hierarchical clustering with Euclidean distance; HDBSCAN, hierarchical density-based spatial clustering of applications with noise; HGOD, outlier detection based on a hybrid graph; HiCS, High Contrast Subspaces; HMM, hidden markov model; HWNN, Hybrid Wavelet Neural Network; iNNE, isolation using nearest neighbor ensembles; INFLO, influenced outlierness; INS, instability factor based outlier detection; IPMOD, information entropy pruning multi-dimensional outlier detection; JBM, Joint bagging-boosting model; KDE, kernel density estimation; KDEOS, Kernel Density Estimation Outlier Score; KLD, Kullback-Leibler Divergence; kmeansTD, k-means target distribution; kNN, k-nearest neighbor; kNN-DTW, k-nearest neighbors dynamic time-warping; Kth-NN, Kth-nearest neighbor; KRXD AD, kernel Reed-Xiaoli detector based anomaly detection; LAD, lipschitz anomaly detector; LDA, linear discriminant analysis; LDCOF, local density cluster-based outlier factor; LDEM, Local density estimation;
LightGBM, Light gradient boosting machine; LOCI, local correlation integral; LDOF, local distance-based outlier factor; LODA, lightweight online detector of anomalies; LOF, local outlier factor;
LOF-UB, local outlier factor upper bound; LoOP, local outlier probability; LogBERT, bidirectional encoder representations from transformers; LS-SVM, Least square support vector machine;
LSCP, locally selective combination of parallel outlier ensembles; LSTM, Long short term memory networks; LUNAR, learnable unified neighborhood-based anomaly ranking; MCD, Minimum Covariance Determinant; MCE, Mean Centred Ellipse; MEB, minimum enclosing ball; MGTD, mixture of gaussian target distribution; MMOD, mini-minimum spanning tree-based outlier detection;
MNN, mutual nearest neighbors; MOGAAL, multiple-objective generative adversarial active learning; MS2OD, outlier detection using minimum spanning tree and medoid selection; MUSC, streaming clustering approach; MVE, High Contrast Subspaces; NaNOD, natural neighbour-based outlier detection; NLOD, novel local outlier detection; NWOD, neighborhood weighted-based outlier detection; OCC, one class classification, OCGP, One Class- Gaussian Processes; OCSVM, one class support vector machine; ODGrCR, outlier detection granular computing and rough set theory; ODIN, outlier detection using in-degree number; OFCOD, on the Fly Clustering Based Outlier Detection; OPTICS, ordering points to identify the clustering structure; PCA, principal component analysis; PB-kNN, pruning-based k-nearest neighbor; PPM, Point Process Models; PSO, particle swarm optimization based anomaly detection; qSp, one-time sampling based anomaly detection; RBF, Radial basis function; RBFN, Radial basis function networks; RDOF, outlier detection based on relative density; RDOS, relative density-based outlier score; RC, Robust Covariance; RCLOS, rough clustering local outlier score; Regression based abnormality detection; RF, Random Forest; RMF, rough membership function based method;RMFB, Rough Membership Function based; RNNs,* *recurrent neural networks; SClust, Spectral Clustering; SGP, Structural Gaussian Process; SGTD, single gaussian target distribution; SOD, subspaces outlier detection; SPiForest, space partition iForest; SLR, Sparse Logistic Regression; SsVGMM, Semi-supervised variational Gaussian mixture model; SOMs, Self-Organizing MapsSubsampling based anomaly detection; SUOD, accelerating large-scale unsupervised heterogeneous outlier Detection; SVDD, support vector data description; SVM, suppose vector machine; TOD, two-stage outlier detection method; uCBLOF, unweighted cluster-based local outlier factor; WDOD, weight density outlier detection; WFRDA, weighted fuzzy-rough density-based anomaly detection; WIF-SGRU, weighted iForest and Siamese Gated recurrent unit; WSVM, weighted support vector machine; XGBoost, eXtreme gradient boosting; XGBOD, extreme gradient boosting outlier detection.*
