# Supplementary material for: Detection of rare medical events in electronic health records using machine learning: Current practices and suggestions – A scoping review
Source: PLoS One. 2026 Mar 16;21(3):e0332963. doi: 10.1371/journal.pone.0332963 (PMC12991209; doi:10.1371/journal.pone.0332963)
Supplement: S7 Table — (DOCX) [file pone.0332963.s008.docx]

**S7 Table: Summary for pairwise comparison between ML algorithms according to ROC AUC**

The table shows the number of times the algorithm in each row outperforms the algorithms in the columns based on the ROC AUC criteria. For example, consider the first row (Ensemble) and the second column (SVM). The value in the intersecting cell is 28, indicating that in studies where both an ensemble algorithm and SVM were used, the ensemble algorithm outperformed SVM 28 times.

|  | **Ensemble** | **SVM** | **LR** | **DL** | **DT** | **kNN** | **NB** |
| --- | --- | --- | --- | --- | --- | --- | --- |
| Ensemble | - | 28 | 20 | 22 | 20 | 10 | 16 |
| SVM | 10 | - | 16 | 16 | 22 | 10 | 12 |
| LR | 22 | 14 | - | 16 | 26 | 10 | 16 |
| DL | 8 | 14 | 14 | - | 22 | 10 | 8 |
| DT | 6 | 8 | 4 | 8 | - | 6 |  |
| kNN | 2 | 2 | 4 | 2 | 6 | - | 4 |
| NB | 6 | 8 | 4 | 6 | 5 | 1 | - |

*DL, Deep Learning; DT, Decision Tree; kNN, k-Nearest Neighbor; LR= Logistic Regression; NB, Naïve Bayes; SVM, Support Vector Machine*
