# Supplementary material for: Detection of rare medical events in electronic health records using machine learning: Current practices and suggestions – A scoping review
Source: PLoS One. 2026 Mar 16;21(3):e0332963. doi: 10.1371/journal.pone.0332963 (PMC12991209; doi:10.1371/journal.pone.0332963)
Supplement: S8 Table — (DOCX) [file pone.0332963.s009.docx]

**S8 Table: the characteristics of the datasets in the included studies**

|  | **Content-Affiliation** | | | | |
| --- | --- | --- | --- | --- | --- |
|  | **Medical- Medical** | **Medical-Methodological** | **Medical-combination** | **Methodological-Methodological** | **Methodological-Combination** |
| n_original | [245, 137774] | [62, 1431597] | [190, 269999] | [48, 1300000] | [50, 9000000] |
| n_ post-under-oversampling |  | [155, 359] | [631, 10172] | [100, 1606] |  |
| n_Anomaly | [9,248] | [4, 116080] | [17, 36528] | [4, 646] | [2, 3547] |
| n_anomaly_post-under-oversampling | [124, 2997] | [0.081, 148] | [160, 5086] | [0.074, 803] | [540573, 540573] |
| P_anomaly_original | [0.00045, 0.087] | [0.004, 0.48] | [0.0012, 0.46] | [0.011, 0.345] | [0.00022, 0.487] |
| P_anomaly_post-under-oversampling |  | [0.125, 0.3454] | [0.029, 0.5] | [0.027, 0.5] | [0.0272, 0.0272] |
| n_features | [5, 50] | [5, 455] | [2, 1841] | [2, 56] | [20, 10000] |
| n_features_ post-feature selection | [2, 2] | [7, 59] | [6, 9485] | [2, 36] | [6, 31] |

*n_original, sample size (number of observations) originally reported; n_anomaly_post-under-oversampling, number of anomalous observations after the application of under/over sampling; n_features, Number of features (variables) before feature selection applied ; n_features_ post-feature selection, Number of features (variables) after feature selection applied; n_anomaly, number of anomalous observations originally reported (before under/over sampling); n_ post-under-oversampling, sample size (number of observations) after under/over sampling applied; P_anomaly_original, Proportion of the anomalous observations before the application of under/over sampling ; P_anomaly_post-under-oversampling, proportion of the anomalous observations after the application of under/over sampling*

*Medical-medical = the study’s primary aim is a medical topic and conducted by a team of researchers with medical expertise or affiliated with medical departments*

*Medical-Methodological = the study’s primary aim is a medical topic and conducted by a team of researchers with expertise in methodology or affiliated with methodology departments*

*Medical-Combination = the study’s primary aim is a medical topic and conducted by a multidisciplinary team of researchers with expertise in medical and methodology, or affiliated with medical and methodology departments*

*Methodological-Methodological = the study’s primary aim is a methodological topic and conducted by a team of researchers with expertise in methodology or affiliated with methodology departments*

*Methodological-Combination = the study’s primary aim is a methodological topic and conducted by a multidisciplinary team of researchers with expertise in medical and methodology, or affiliated with medical and methodology departments*
