# Supplementary material for: Detection of rare medical events in electronic health records using machine learning: Current practices and suggestions – A scoping review
Source: PLoS One. 2026 Mar 16;21(3):e0332963. doi: 10.1371/journal.pone.0332963 (PMC12991209; doi:10.1371/journal.pone.0332963)
Supplement: S9 Table — (DOCX) [file pone.0332963.s010.docx]

**S9 Table: The metrics used to evaluate the anomaly detection algorithms (total count) by the included studies**

|  | **Content-Affiliation** | | | | |
| --- | --- | --- | --- | --- | --- |
|  | **Medical- Medical** | **Medical-Methodological** | **Medical-combination** | **Methodological-Methodological** | **Methodological-Combination** |
| Median # of metrics | 2 | 4 | 4 | 2 | 1 |
| Minimum # of metrics | 1 | 1 | 1 | 1 | 1 |
| Maximum # of metrics | 5 | 7 | 7 | 6 | 4 |
| Confusion matrix presented | 1 | 5 | 2 | 1 |  |
| AUC or C-statistic | 3 | 9 | 30 | 19 | 8 |
| AUPRC |  | 0 | 2 | 3 |  |
| F1 score or F1 measure | 1 | 7 | 12 | 6 | 1 |
| Recall or sensitivity or TPR |  | 10 | 19 | 16 | 3 |
| Precision |  | 6 | 9 | 11 | 2 |
| Accuracy |  | 9 | 16 | 14 | 2 |
| FNR |  | 0 | 1 |  |  |
| Specificity |  | 4 | 11 | 4 | 2 |
| NPV |  | 1 | 3 | 2 |  |
| PPV |  | 1 | 2 | 1 |  |
| FDR or FPR or FAR |  |  |  |  |  |
| Youden |  |  |  |  |  |
| Kappa |  | 1 |  | 1 |  |
| BS |  |  | 3 |  | 1 |
| MCC |  | 1 | 2 |  |  |
| HMSS |  |  | 1 |  |  |
| BER |  |  | 1 |  |  |
| GM |  |  | 1 | 1 |  |

*# = number, AUC, Area under curve, AUCPR, Area under precision recall curve, BER, Balanced error rate; BC, Brier score; FAR, False alarm rate; FDR, False discovery rate, FNR, False negative rate; FPR, False positive rate; GM, Geometric mean; HMSS, Harmonic mean for subspace selection; MCC, Matthews Correlation Coefficient; NPV, negative predictive value; PPV, positive predictive value; TPR, True positive rate*

*Medical-medical = the study’s primary aim is a medical topic and conducted by a team of researchers with medical expertise or affiliated with medical departments*

*Medical-Methodological = the study’s primary aim is a medical topic and conducted by a team of researchers with expertise in methodology or affiliated with methodology departments*

*Medical-Combination = the study’s primary aim is a medical topic and conducted by a multidisciplinary team of researchers with expertise in medical and methodology, or affiliated with medical and methodology departments*

*Methodological-Methodological = the study’s primary aim is a methodological topic and conducted by a team of researchers with expertise in methodology or affiliated with methodology departments*

*Methodological-Combination = the study’s primary aim is a methodological topic and conducted by a multidisciplinary team of researchers with expertise in medical and methodology, or affiliated with medical and methodology departments*
