# Supplementary material for: Detection of rare medical events in electronic health records using machine learning: Current practices and suggestions – A scoping review
Source: PLoS One. 2026 Mar 16;21(3):e0332963. doi: 10.1371/journal.pone.0332963 (PMC12991209; doi:10.1371/journal.pone.0332963)
Supplement: S10 Table — (DOCX) [file pone.0332963.s011.docx]

**S10 Table: The metrics used as sole performance indicators in the included studies**

|  | **Content-Affiliation** | | | | |
| --- | --- | --- | --- | --- | --- |
|  | **Medical- Medical** | **Medical-Methodological** | **Medical-combination** | **Methodological-Methodological** | **Methodological-Combination** |
| AUC or c-statistic | 1 | 3 | 6 | 9 | 6 |
| AUPRC |  |  |  | 2 |  |
| F1 |  |  |  | 1 |  |
| Recall |  |  |  | 3 |  |

*AUC, Area under curve; AUCPR, Area under precision recall curve*

*Medical-medical = the study’s primary aim is a medical topic and conducted by a team of researchers with medical expertise or affiliated with medical departments*

*Medical-Methodological = the study’s primary aim is a medical topic and conducted by a team of researchers with expertise in methodology or affiliated with methodology departments*

*Medical-Combination = the study’s primary aim is a medical topic and conducted by a multidisciplinary team of researchers with expertise in medical and methodology, or affiliated with medical and methodology departments*

*Methodological-Methodological = the study’s primary aim is a methodological topic and conducted by a team of researchers with expertise in methodology or affiliated with methodology departments*

*Methodological-Combination = the study’s primary aim is a methodological topic and conducted by a multidisciplinary team of researchers with expertise in medical and methodology, or affiliated with medical and methodology departments*
