# Supplementary material for: Detection of rare medical events in electronic health records using machine learning: Current practices and suggestions – A scoping review
Source: PLoS One. 2026 Mar 16;21(3):e0332963. doi: 10.1371/journal.pone.0332963 (PMC12991209; doi:10.1371/journal.pone.0332963)
Supplement: S11 Table — (DOCX) [file pone.0332963.s012.docx]

**S11 Table: Summary of the categorical features (frequency) among the extracted features**

|  | | **Content-Affiliation** | | | | |
| --- | --- | --- | --- | --- | --- | --- |
|  | | **Medical- Medical** | **Medical-Methodological** | **Medical-combination** | **Methodological-**  **Methodological** | **Methodological-Combination** |
| Reported limitations | Data related | 2 | 6 | 17 | 3 | 3 |
|  | Methodological related |  |  | 9 | 4 | 10 |
|  | Application related |  | 1 | 1 | 2 |  |
| Resampling | All used |  | 7 | 12 | 6 |  |
|  | under sampling (Random) |  | 2 | 4 | 1 |  |
|  | oversampling (random) |  | 1 | 3 | 1 |  |
|  | SMOTE |  | 4 | 6 | 3 |  |
|  | SMOTEtomek |  |  | 1 | 2 |  |
|  | ADASYN |  |  | 3 | 0 |  |
|  | Other |  |  |  | 2 |  |
| Data accessibility | Open | 1 | 12 | 15 | 40 | 7 |
|  | Protected | 3 | 6 | 23 | 6 | 4 |
| Data split | Reported | 3 | 18 | 36 | 21 | 4 |
|  | Train/test | 1 | 8 | 14 | 6 | 2 |
|  | Train/test + 5 folds cross validation |  |  | 3 | 1 | 1 |
|  | Train/test + 10folds cross validation |  |  | 7 | 5 | 1 |
|  | 5 folds cross validation | 1 | 6 | 3 | 2 |  |
|  | 10 folds cross validation | 1 | 3 | 7 | 5 |  |
|  | Train/test/validation |  | 1 | 1 | 1 |  |
|  | Cross validation (folds not reported) |  |  | 1 | 1 |  |
| Biologically implausible values detection | Reported | 1 | 2 | 3 | 5 | 1 |
|  | Removed (without mentioning the technique) |  | 1 | 1 | 2 |  |
|  | Threshold (author defined, standard deviation based) | 1 |  | 1 |  |  |
|  | OCSVM |  | 1 |  |  |  |
|  | Moving average |  |  | 1 |  |  |
|  | DBSCAN |  |  |  | 1 |  |
|  | iForest |  |  |  | 1 |  |
|  | k-means |  |  |  | 1 |  |
|  | Boxplot + LOF |  |  |  |  | 1 |
| Scaling | Reported | 1 | 11 | 12 | 17 | 3 |
|  | Normalized (mean =0, standard deviation=1) | 1 | 5 | 8 | 9 | 3 |
|  | Min-max | 0 | 5 | 3 | 7 | 0 |
|  | Other |  | 1 | 1 | 1 |  |
| Missing imputation | Reported | 2 | 12 | 30 | 10 | 2 |
|  | Removed | 2 | 3 | 6 | 4 |  |
|  | Mean/median/mode |  | 4 | 7 | 5 | 1 |
|  | k-nearest neighbor |  | 1 | 1 | 1 | 0 |
|  | MICE |  | 1 | 2 |  | 0 |
|  | missForst |  | 1 | 1 |  | 1 |
|  | Random forest regressor |  | 1 | 1 |  |  |
|  | Missing category created |  | 1 | 4 |  |  |
|  | K means |  |  | 1 |  |  |
| Papers that consider missing mechanism | |  | 0 | 3 |  | 1 |
| Feature selection (reported) | | 2 | 10 | 27 | 14 | 2 |
| Algorithm | Supervised | 2 | 15 | 31 | 11 | 4 |
|  | Unsupervised | 2 | 3 | 9 | 32 | 6 |
|  | Semi-supervised |  |  |  | 1 | 1 |

*ADASYN, Adaptive synthetic; LOF, Local outlier factor; MICE, Multivariate Imputation by Chained Equations; OCSVM, One class support vector machine; SMOTE, Synthetic Minority Oversampling Technique;* SMOTEtomek , synthetic Minority Oversampling Technique and Tomek links

*Medical-medical = the study’s primary aim is a medical topic and conducted by a team of researchers with medical expertise or affiliated with medical departments*

*Medical-Methodological = the study’s primary aim is a medical topic and conducted by a team of researchers with expertise in methodology or affiliated with methodology departments*

*Medical-Combination = the study’s primary aim is a medical topic and conducted by a multidisciplinary team of researchers with expertise in medical and methodology, or affiliated with medical and methodology departments*

*Methodological-Methodological = the study’s primary aim is a methodological topic and conducted by a team of researchers with expertise in methodology or affiliated with methodology departments*

*Methodological-Combination = the study’s primary aim is a methodological topic and conducted by a multidisciplinary team of researchers with expertise in medical and methodology, or affiliated with medical and methodology departments*
