# Supplementary material for: Detection of rare medical events in electronic health records using machine learning: Current practices and suggestions – A scoping review
Source: PLoS One. 2026 Mar 16;21(3):e0332963. doi: 10.1371/journal.pone.0332963 (PMC12991209; doi:10.1371/journal.pone.0332963)
Supplement: S12 Table — (DOCX) [file pone.0332963.s013.docx]

**S12 Table: Example of adjusting machine learning output to control false positives and negatives**

|  | | **Threshold 0.5** | | **Threshold 0.3** | | **Threshold 0.7** | |
| --- | --- | --- | --- | --- | --- | --- | --- |
|  |  | **Actual** | | **Actual** | | **Actual** | |
|  |  | **Positive** | **Negative** | **Positive** | **Negative** | **Positive** | **Negative** |
| Predicted | Positive | 155 | 39 | 150 | 23 | 147 | 18 |
|  | negative | 9 | 125 | 14 | 141 | 17 | 146 |

Consider an algorithm that outputs a probability score between 0 and 1, reflecting the likelihood of a certain disease. For illustration, consider the clinical dataset provided by the Albert Einstein Israelite Hospital in Sao Paulo, Brazil available via the link   [https://www.kaggle.com/einst eindata4u/covid19](https://www.kaggle.com/einst%20eindata4u/covid19) (the dataset includes the presence or absence of a diagnosis of COVID-19 and 34 potential predictor variables from 1,091 patients after preprocessing). If a logistic regression algorithm is used to diagnose Covid-19, at the default cutoff between predicting an outcome of ‘diagnosis present’ or ‘diagnosis absent’ (0.5) for the test set, 24 out of 328 patients would receive a false positive COVID-19 diagnosis and 9 patients a false negative one. Here, false negative could result in increased risk of transmission and delayed treatment while false positive could result in psychological stress and strain on healthcare resources. Depending on the preferred balance between false positives and false negatives, we could either (1) lower the threshold (e.g., to 0.3), causing a higher number of false positives (39) but lower number of false negatives (9), or (2) raise the threshold (e.g., to 0.7), causing fewer false positives (18) but more false negatives (17).
